# A common progenitor gives rise to fibroblastic reticular cells and vascular smooth muscle cells in murine lymph nodes

**DOI:** 10.1084/jem.20242300

**Published:** 2025-11-26

**Authors:** Lisa Kurz, Mechthild Lütge, Angelina De Martin, Hung-Wei Cheng, Elina Bugar, Yves Stanossek, Samuel Meili, Joshua D. Brandstadter, Ivan Maillard, Lucas Onder, Burkhard Ludewig

**Affiliations:** 1 Institute of Immunobiology, Cantonal Hospital St. Gallen, St. Gallen, Switzerland; 2 Department of Otorhinolaryngology, Head and Neck Surgery, Cantonal Hospital St. Gallen, St. Gallen, Switzerland; 3 Division of Hematology-Oncology, Department of Medicine, University of Pennsylvania Perelman School of Medicine, Philadelphia, PA, USA; 4 Division of Hematologic Malignancies, Department of Medicine & Human Oncology and Pathogenesis Program, Memorial Sloan Kettering Cancer Center, New York, NY, USA; 5 University Heart Center, University Hospital Zurich and University of Zurich, Zurich, Switzerland; 6 Center for Translational and Experimental Cardiology, University Hospital Zürich and University of Zürich, Zürich, Switzerland

## Abstract

The interaction of immune cells in the lymph node microenvironment depends on the infrastructure and molecular cues provided by fibroblastic reticular cells (FRCs). In addition, concentric layers of still poorly defined mural cells, including vascular smooth muscle cells (VSMCs), are involved in positioning and regulating immune cell interactions in different lymph node compartments. Using time-resolved single-cell transcriptomics, combined with cell fate mapping and high-resolution confocal microscopy, we found that lymph node FRCs and VSMCs share a proliferating, CCL19-expressing embryonic progenitor. Trajectory analysis identified lymphotoxin β receptor (LTβR)-dependent lineages that gave rise to FRCs underpinning the subcapsular sinus, T and B cell zones, and the medulla. LTβR-independent development of VSMCs and perivascular reticular cells from the common progenitor highlighted the close developmental relationship between FRCs and mural cells. Collectively, these results indicate that CCL19-expressing perivascular progenitors are capable of generating the fibroblastic and mural cell infrastructure of murine lymph nodes.

## Teaser

The study by Kurz et al. elucidates the ontogeny of fibroblastic reticular cells and vascular smooth muscle cells in mouse lymph nodes from proliferating, CCL19-expressing progenitors and highlights the close lineage relationship of the progeny in the perivascular niche.

## Introduction

Lymph nodes serve as immune surveillance hubs for peripheral tissues and internal organs by channeling extracellular fluids through immune cell compartments that secure antigen sampling and subsequent activation and regulation of innate and adaptive immune responses (Acton et al., 2021; Onder and Ludewig, 2018). The strategic localization of lymph nodes at intersections of the lymphatic and blood vasculature requires close interaction between endothelial and fibroblastic stromal cells during development and adulthood to control immune cell function (Bovay et al., 2018; Onder et al., 2013; Onder et al., 2017; Onder et al., 2012). Lymphocytes and myeloid cells leave the blood stream at dedicated exit points of the vasculature to enter the lymph node parenchyma via high endothelial venules (HEVs) (Anderson and Anderson, 1976; Ugur et al., 2023). The parenchyma of all organs, including lymph nodes, is separated from the bloodstream by an endothelial barrier that is underpinned by concentric layers of mural cells (i.e., pericytes and vascular smooth muscle cells [VSMCs]) (Lütge et al., 2025; Muhl et al., 2020; Muhl et al., 2022; Vanlandewijck et al., 2018). However, the origin of mural cells and their relationship to other fibroblastic stromal cells in the lymph node are still poorly understood.

Early morphological studies on lymphocyte migration in rat lymph nodes and Peyer’s patches have characterized the fibroblastic stromal cells in the perivascular space of HEVs as “overlapping reticular cell plates” (Anderson et al., 1976). Lymphoid organ fibroblasts with specific functions for establishing immune cell niches are referred to as fibroblastic reticular cells (FRCs) (De Martin et al., 2024; Gretz et al., 1997; Krishnamurty and Turley, 2020). Single-cell transcriptomics analysis has enabled identification and comprehensive characterization of specialized FRC subsets in mice and humans, including perturbation-mediated changes of their activation state (Alexandre et al., 2022; De Martin et al., 2023; Lütge et al., 2025; Mourcin et al., 2021; Perez-Shibayama et al., 2020; Prados et al., 2021; Rodda et al., 2018). CXCL13-producing B cell zone reticular cell (BRC) subsets (Cosgrove et al., 2020; Pikor et al., 2020) include marginal reticular cells (MRCs) in the subcapsular sinus, where they provide niches for macrophages and dendritic cells and thereby facilitate the establishment of an antigen sampling zone (Camara et al., 2019; Lütge et al., 2023). Follicular dendritic cells (FDCs) in the light and dark zone of the germinal center together with T-B cell zone reticular cells (TBRCs) control activation and differentiation of B cells and their interaction with T helper cells (Pikor et al., 2020; Rodda et al., 2015). T cell zone reticular cells (TRCs) support T cell–dendritic cell interaction through the provision of the chemokines CCL19 and CCL21 and provide cytokines such as IL-7 (Knop et al., 2020; Link et al., 2007). FRCs in the deep paracortex and medulla that interact with various immune cells, including plasma cells, are categorized as medullary reticular cells (MedRCs) (Dasoveanu et al., 2020; Huang et al., 2018). These specialized FRC subsets are physically connected to still poorly defined mural cells of the lymph node vasculature by a layer of perivascular reticular cells (PRCs) (De Martin et al., 2023; Lütge et al., 2023; Lütge et al., 2025; Novkovic et al., 2020). It is thus important to define the molecular characteristics of fibroblastic stromal cell populations in different lymph node environments and to determine the developmental pathways that facilitate the formation of distinct functional niches for immune cells.

Here, we employed cell fate-mapping and lineage-tracing models based on time-resolved Cre recombinase activity in *Ccl19*- and *Cxcl13*-expressing cells to define the origins and differentiation trajectories of lymph node FRCs and mural cells. We found that both FRCs and VSMCs are generated from a perivenular CCL19-expressing embryonic progenitor. Development of specialized FRCs was lymphotoxin β receptor (LTβR)-dependent, whereas VSMC and PRC differentiation from the common progenitor was LTβR-independent. In sum, these findings demonstrate the close relationship between FRCs and mural cells and show that a shared perivascular progenitor can develop into all types of fibroblastic stromal cells in mouse lymph nodes.

## Results

### Genetic targeting of FRCs and VSMCs in murine lymph nodes

**Figure S1.**
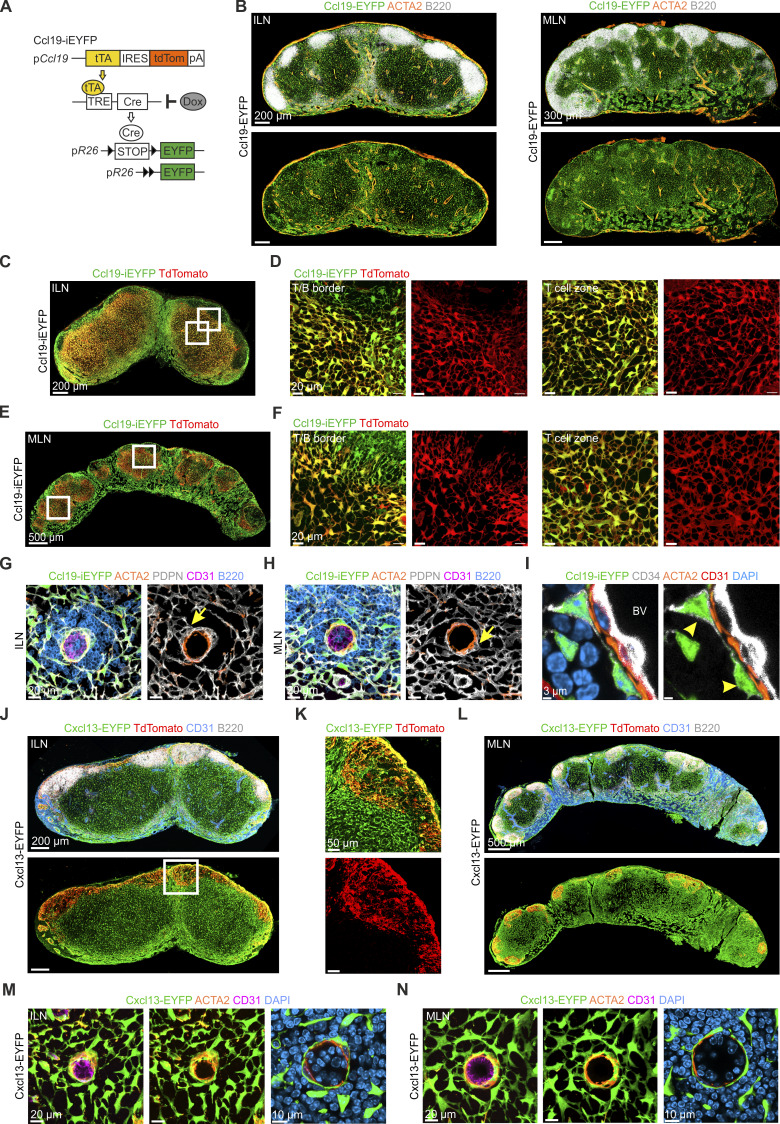
**Transgene activity in peripheral and mesenteric lymph nodes of Ccl19-iEYFP and Cxcl13-EYFP mice. (A)** Schematic representation of the triple transgenic Ccl19-iEYFP mouse model. **(B)** Confocal microscopy images of cross sections of representative inguinal and mesenteric lymph nodes from 8-wk-old Ccl19-Cre (Ccl19-EYFP) mice. Microscopy images are representative for three inguinal and three mesenteric lymph nodes from two independent experiments. Scale bars: 200 µm (left panels) and 300 µm (right panels). **(C–F)** Confocal microscopy images showing current *Ccl19* promoter activity (TdTomato expression) in cross sections of inguinal (C and D) and mesenteric lymph nodes (E and F) from Ccl19-iEYFP mice. Boxes indicate regions of higher magnification. Microscopy images are representative for three inguinal and three mesenteric lymph nodes from two independent experiments. Scale bars: 200 µm, 500 µm (C and E, overviews) and 20 µm (D and F, zoom-ins). **(G and H)** Confocal microscopy images showing venules at higher magnification in inguinal (G) and mesenteric (H) lymph nodes from Ccl19-iEYFP mice. Arrows indicate the connection of VSMCs to the FRC network by PDPN^+^ PRCs. Microscopy images are representative for three inguinal and three mesenteric lymph nodes from two independent experiments. Scale bars: 20 µm. **(I)** Confocal microscopy images showing blood vessels (BV) at high magnification surrounded by ACTA2^+^ VSMCs and CD34^+^ PRCs. **(J–N)** Confocal microscopy images of inguinal and mesenteric lymph nodes from Cxcl13-EYFP mice. **(J)** Confocal microscopy images of cross sections of representative inguinal lymph nodes from 8-wk-old Cxcl13-EYFP mice. **(K)** Confocal microscopy images of higher magnified B cell follicles with TdTomato indicating CXCL13 expression. **(L)** Confocal microscopy images of cross sections of representative mesenteric lymph nodes from 8-wk-old Cxcl13-EYFP mice. **(M and N)** Confocal microscopy images showing venules at higher magnification in inguinal (M) and mesenteric lymph nodes (N) from Cxcl13-EYFP mice. Microscopy images are representative for three inguinal and three mesenteric lymph nodes from three independent experiments. Overviews in B, C, E, J, and L are stitched images from tile scans. Scale bars: 200 µm (J), 50 µm (K), 500 µm (L), 20, and 10 µm (M and N).

**Figure 1.**
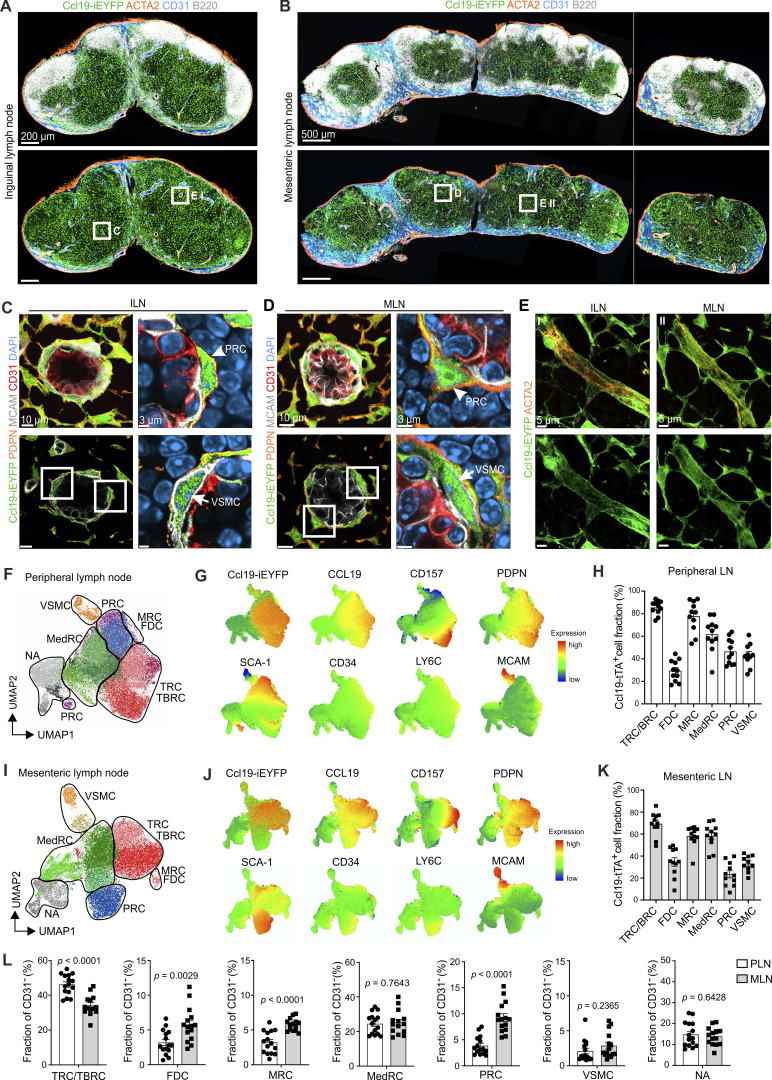
**CCL19-expressing FRCs and VSMCs in murine lymph nodes. (A and B)** Confocal microscopy images showing cross sections of inguinal lymph nodes and the mesenteric lymph node chain from Ccl19-iEYFP^+^ mice. Boxed areas indicate regions of higher magnification micrographs. Microscopy images are stitched tile scans representative for four inguinal and four mesenteric lymph nodes from three independent experiments. Scale bars: 200 µm (A) and 500 µm (B). **(C–E)** Confocal microscopy images showing the blood vessels in the inguinal and mesenteric lymph nodes at higher magnification. Arrows and arrowheads highlight the localization of VSMC and PRC around the vessel. High-resolution images are representative for three inguinal and three mesenteric lymph nodes from three independent experiments. Scale bars: 10 µm (C and D, left panels), 3 µm (C and D, right panels), and 5 µm (E). **(F–L)** Flow cytometric analysis of non-hematopoietic cells in peripheral (inguinal, axillary, and brachial) and mesenteric lymph nodes. **(F and I)** Phenograph clustering projected on UMAP showing CD31^−^ cells from pooled lymph nodes. **(G and J)** Expression of surface markers used to identify different FRC and VSMC populations projected on the UMAP. **(H and K)** Quantification of Ccl19-iEYFP^+^ cells gated according to the gating strategy shown in Fig. S2, A and C with pre-gating on CD31^−^ cells. Data are shown as the mean and SEM from *n* = 11 mice from three independent experiments. **(L)** Quantification of the relative abundance of different FRC and VSMC populations. Relative abundances were calculated according to the gating strategy shown in Fig. S2, A and C, and data are shown as the mean and SEM from *n* = 15 mice from four independent experiments. P values were calculated with unpaired Student’s *t* test.

Doxycycline (Dox)-regulated expression of the tetracycline transactivator (tTA) in *Ccl19*-expressing cells permits dissection of FRC progenitor-progeny relationship in secondary lymphoid organs and in tertiary lymphoid structures in tumors (Cheng et al., 2022; Cheng et al., 2019; Onder et al., 2025; Prados et al., 2021). The temporal regulation of Cre recombinase activity labeled FRC precursors and progeny in situ when crossed to mice that facilitate Cre recombinase–dependent expression of the enhanced yellow fluorescent protein (EYFP), with the resulting strain abbreviated as Ccl19-iEYFP (Fig. S1 A). In the absence of Dox application, the Ccl19-iEYFP model traces a lineage of lymph node FRCs that was found across all compartments of inguinal and mesenteric lymph nodes of adult mice (Fig. 1, A and B), thus phenocopying the Cre recombinase activity and FRC lineage-tracing pattern found in the constitutive Ccl19-Cre model (Chai et al., 2013; Perez-Shibayama et al., 2020) (Fig. S1 B). In addition to lineage-tracing via Cre recombinase expression in Ccl19-iEYFP mice, the co-expression of the red fluorescent protein TdTomato facilitated assessment of current *Ccl19* promoter activity, which was highest in the T cell zone and T/B border of both inguinal (Fig. S1, C and D) and mesenteric lymph nodes (Fig. S1, E and F). The analysis of EYFP expression in different perivascular areas using high-resolution confocal microscopy revealed the presence of Ccl19-tTA^+^ MCAM^+^ VSMCs (Fig. 1, C and D, arrows) and Ccl19-tTA^+^ PDPN^+^ PRCs (Fig. 1, C and D, arrowheads) around HEVs. Additional staining for the classical lymph node FRC marker podoplanin (PDPN) confirmed that PDPN^+^ PRCs provide a direct physical link between VSMCs and the wider FRC network around HEVs (Fig. S1, G and H, arrows). The two adjacent layers of VSMCs and PRCs targeted by the Ccl19-tTA transgene were also detectable around smaller blood vessels, such as parenchymal venules (Fig. 1 E). As *Ccl19* expression in BRCs is regionally restricted (Lütge et al., 2023; Rodda et al., 2018), we complemented our analysis using Cxcl13-Cre/TdTomato R26R-EYFP mice (abbreviated as Cxcl13-EYFP), which facilitate lineage tracing of CXCL13-expressing progenitors in the early lymph node anlage (Onder et al., 2017) and genetically mark all BRC subsets in adult lymph nodes (Lütge et al., 2023; Pikor et al., 2020). Histological analysis of adult inguinal and mesenteric lymph nodes confirmed the broad expression of the Cxcl13-EYFP transgene in lymph node FRCs (Fig. S1, J–L). In addition, dense networks of Cxcl13-Cre lineage-traced PRCs were connected to EYFP^+^ VSMCs in inguinal and mesenteric lymph nodes (Fig. S1, M and N).

**Figure S2.**
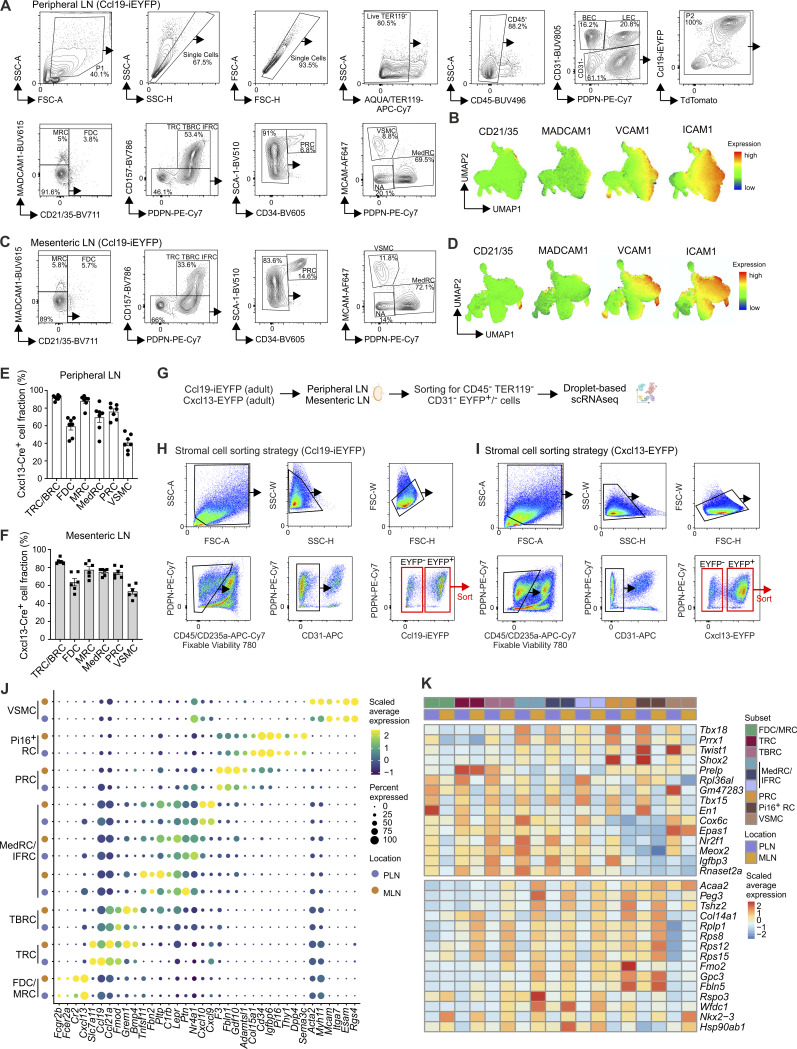
**Flow cytometric and single-cell transcriptomic analysis of FRCs and VSMCs in peripheral and mesenteric lymph nodes. (A–D)** Representative flow cytometric analysis of peripheral (A and B) and mesenteric (C and D) lymph nodes of 8-wk-old Ccl19-iEYFP mice. Manual gating strategy used for gating of FRC and VSMC populations in peripheral (A) and mesenteric (C) lymph nodes. Relative expression of the indicated surface markers projected on the UMAP representation of FRC and VSMC populations of peripheral (B) and mesenteric (D) lymph nodes. Data are representative for *n* = 15 mice from four independent experiments (A and C) and *n* = 11 mice from three independent experiments (B and D). **(E and F)** Quantification of Cxcl13-EYFP^+^ cells gated according to the gating strategy shown in Fig. S2, A and C with pre-gating on CD31^−^ cells. Data are shown as the mean and SEM from *n* = 7 (E) and *n* = 6 mice (F) from two independent experiments. **(G)** Schematic representation of the experimental approach used for the single-cell transcriptomics analysis. **(H)** Representative sorting strategy of non-hematopoietic cells isolated from peripheral and mesenteric lymph nodes of Ccl19-iEYFP mice. **(I)** Representative sorting strategy of non-hematopoietic cells isolated from peripheral and mesenteric lymph nodes of Cxcl13-EYFP mice. **(J)** Dot plot showing the average expression of signature genes in FRC subsets and VSMCs isolated from Ccl19-iEYFP mice compared between peripheral and mesenteric lymph nodes. **(K)** Top 15 differentially expressed genes upregulated in FRC subsets and VSMCs from peripheral and mesenteric lymph nodes. Lymph node scRNA-seq data from Ccl19-iEYFP is representative of *n* = 15 mice from four independent experiments. Figure was supplemented with elements from https://BioRender.com.

Next, we used multicolor flow cytometry to quantitatively assess the composition of non-hematopoietic, CD31^–^ cells in peripheral (pooled inguinal, axillary, and brachial) and mesenteric lymph nodes. Using a combination of established lymph node FRC and VSMC markers, in addition to TdTomato as proxy for current CCL19 expression, all major FRC populations and VSMCs were detectable in peripheral (Fig. 1, F–H; and Fig. S2, A and B) and mesenteric lymph nodes (Fig. 1, I–K; and Fig. S2, C and D). As expected, PDPN and CCL19 expression was found across different FRC populations, with TRC/TBRC clusters showing the highest expression of these markers in uniform manifold approximation and projection (UMAP) clustering (Fig. 1, G and J). PRCs could be distinguished by CD34, SCA-1, and LY6C expression (Fig. 1, G–J; and Fig. S1 I), whereas the adhesion molecule MCAM was found to be an exclusive marker for VSMCs (Fig. 1, G and J). Isolation and generation of single-cell suspensions of MRCs and FDCs is challenging due the high abundance of veil-like cell membrane protrusions that provide large surface interaction areas with immune cells (Cosgrove et al., 2020; Martínez-Riaño et al., 2023). The digestion and stromal cell separation protocol used here facilitated assessment of CD21/35 and MADCAM1 expression to distinguish MRCs and FDCs (Fig. S2, B and D) and to determine Ccl19-tTA–dependent Cre recombinase expression (Ccl19-tTA^+^) also in these rare FRC subsets (Fig. 1, H and K). Likewise, flow cytometric analysis revealed Cxcl13-Cre activity in all FRC subsets and VSMCs in peripheral and mesenteric lymph nodes from Cxcl13-EYFP mice (Fig. S2, E and F). Classification and quantification of FRC subsets (Fig. S2, A and C) showed that TRCs/TBRCs and MedRCs were most abundant both in peripheral and mesenteric lymph nodes (Fig. 1 L). The frequency of PRCs was consistently higher than the frequency of VSMCs in both types of lymph nodes (Fig. 1 L and Fig. S2, A and C). These data demonstrate that EYFP labeling in the Ccl19-iEYFP mouse model is well suited for the delineation of FRC and mural cell differentiation trajectories in murine lymph nodes.

### Single-cell transcriptomics–based characterization of the FRC and mural cell landscape

**Figure 2.**
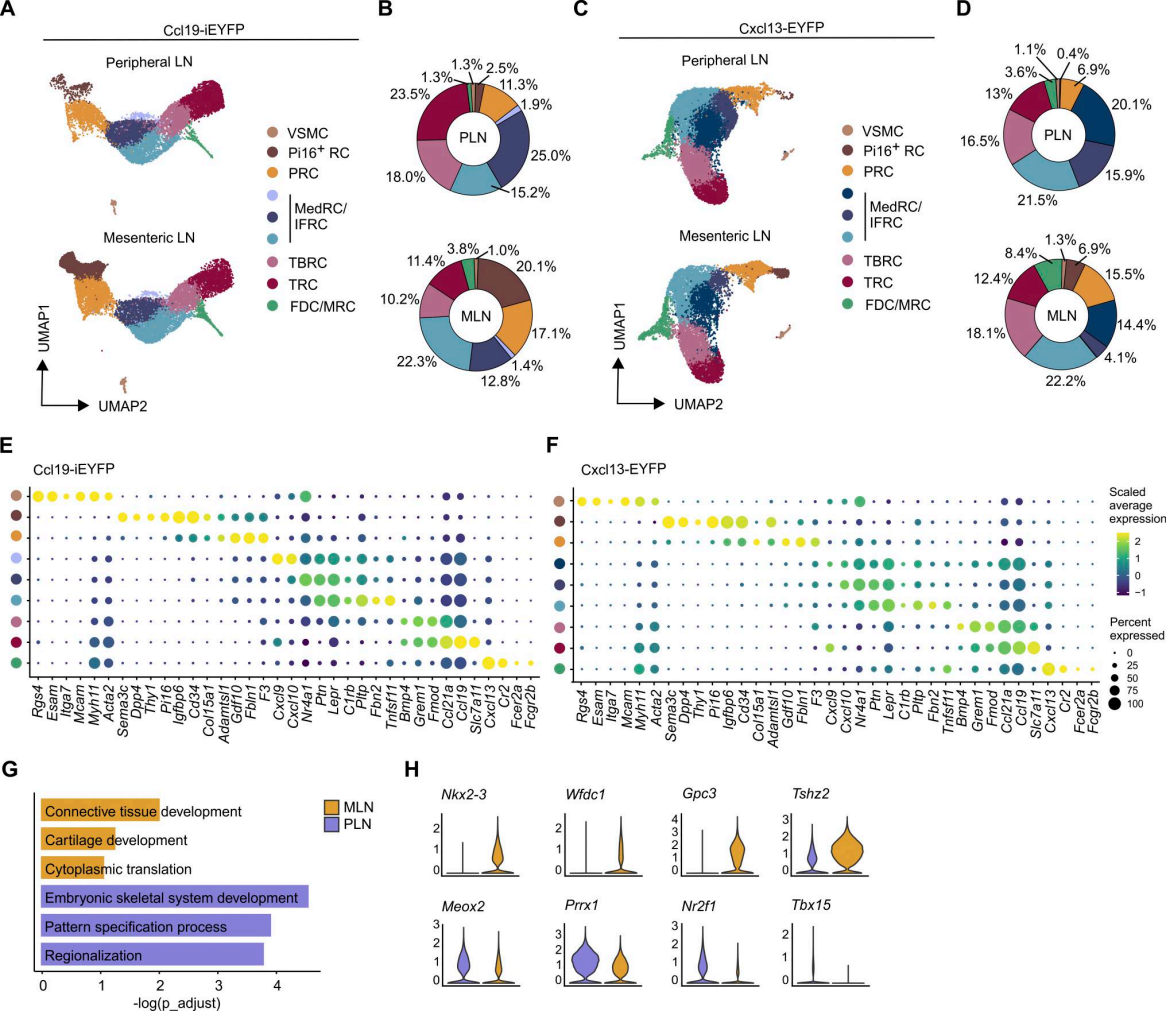
**Molecular characterization of lymph node FRCs and VSMCs. (A and B)** scRNA-seq data of VSMCs and FRCs from peripheral and mesenteric lymph nodes of 8-wk-old Ccl19-iEYFP mice. **(A)** UMAP representation split by lymph node location and colored by subset identity. **(B)** Relative abundance of FRC subsets and VSMCs in peripheral and mesenteric murine lymph nodes of Ccl19-iEYFP mice. **(C and D)** scRNA-seq data of VSMCs and FRCs from peripheral and mesenteric lymph nodes of 8-wk-old Cxcl13-EYFP mice. **(C)** UMAP representation split by lymph node location and colored by subset identity. **(D)** Relative abundance of FRC subsets and VSMCs in peripheral and mesenteric murine lymph nodes of Cxcl13-EYFP mice. **(E)** Dot plot indicating the average expression of signature genes across VSMCs and FRC subsets in lymph nodes of Ccl19-iEYFP mice. **(F)** Dot plot indicating the average expression of signature genes across VSMCs and FRC subsets in lymph nodes of Cxcl13-EYFP mice. **(G and H)** Differentially expressed gene analysis between FRCs and VSMCs isolated from peripheral and mesenteric lymph nodes. **(G)** Enriched gene sets based on differentially expressed genes in peripheral and mesenteric lymph nodes. **(H)** Violin plots showing gene expression profiles of selected differentially expressed genes. Lymph node scRNA-seq data of Ccl19-iEYFP mice are representative of *n* = 15 mice from four independent experiments; 52,188 cells in total. Lymph node scRNA-seq data of Cxcl13-EYFP mice are representative of *n* = 10 mice from two independent experiments; 22,288 cells in total.

To elaborate the molecular characteristics of lymph node FRCs and VSMCs, we performed single-cell RNA sequencing (scRNA-seq) analyses of sorted CD45^−^ Ter119^−^ CD31^−^ cells from peripheral and mesenteric lymph nodes of adult Ccl19^−^iEYFP and Cxcl13-EYFP mice (Fig. S2, G–I). The detailed characterization of non-endothelial (CD31^−^) stromal cells, based on established lymph node FRC markers (Lütge et al., 2023; Perez-Shibayama et al., 2020; Rodda et al., 2018), revealed the presence of eight FRC clusters and one VSMC cluster in peripheral and mesenteric lymph nodes (Fig. 2, A–D). Consistent with the flow cytometric analysis (Fig. 1 L), TRCs and TBRCs represented large FRC populations characterized by high expression of *Ccl19*, *Ccl21a*, and *Grem1* (Fig. 2, B and D–F). Three clusters of MedRCs and interfollicular reticular cells (IFRCs) could be distinguished by *Nr4a1*, *Lepr*, and *Tnfsf11* expression, while the shared FDC/MRC cluster showed highest expression of *Cxcl13* among all FRC subsets (Fig. 2, E and F). Based on established perivascular cell markers (Vanlandewijck et al., 2018), we identified a cluster of VSMCs with high expression of vascular adhesion molecule *Mcam* and the smooth muscle proteins *Acta2* and *Myh11* (Fig. 2, E and F). In addition, two clusters with shared expression of *Cd34* were identified as PRCs and peptidase inhibitor-16–expressing reticular cells (Pi16^+^ RCs) (Fig. 2, E and F). PRCs expressed genes involved in extracellular matrix (ECM) organization, such as *Fbln1*, *Col15A1*, and *Gdf10*, while the distinction of Pi16^+^ RCs from other FRC subsets is based on the expression of *Pi16*, *Dpp4*, and the highest *Cd34* expression among all FRC subsets (Fig. 2, E and F) (Buechler et al., 2021; Lütge et al., 2023). The comparison of the transcriptional profiles of FRCs and VSMCs isolated from peripheral and mesenteric lymph nodes revealed a comparable expression of marker genes in both types of lymph nodes (Fig. S2 J). To further assess whether the specific location of lymph nodes in the body and thus differences in their developmental origin affect organ-specific gene signatures, we employed gene set enrichment analysis based on the top 15 differentially expressed genes in FRC subsets and VSMCs of peripheral and mesenteric lymph nodes (Fig. S2 K). Gene sets specific to mesenteric lymph node FRCs included genes involved in connective tissue development, cartilage development, and cytoplasmic translation (Fig. 2 G), whereas FRCs from peripheral lymph nodes showed enrichment of gene sets involved in embryonic skeletal development and pattern specification processes (Fig. 2 G). In addition, differential expression of location-specific transcription factors and transcriptional regulators pointed toward anatomical and structural imprints in the transcriptome of lymph node FRCs and VSMCs (Fig. 2 H and Fig. S2 K). Overall, these data confirm that FRC subset identity is conserved across lymphoid organs (De Martin et al., 2024; Lütge et al., 2023) and that the key functional features of Ccl19-tTA lineage-traced FRCs and VSMCs are maintained in peripheral and mesenteric lymph nodes.

### Differentiation of FRCs and VSMCs from CCL19-expressing progenitors

**Figure 3.**
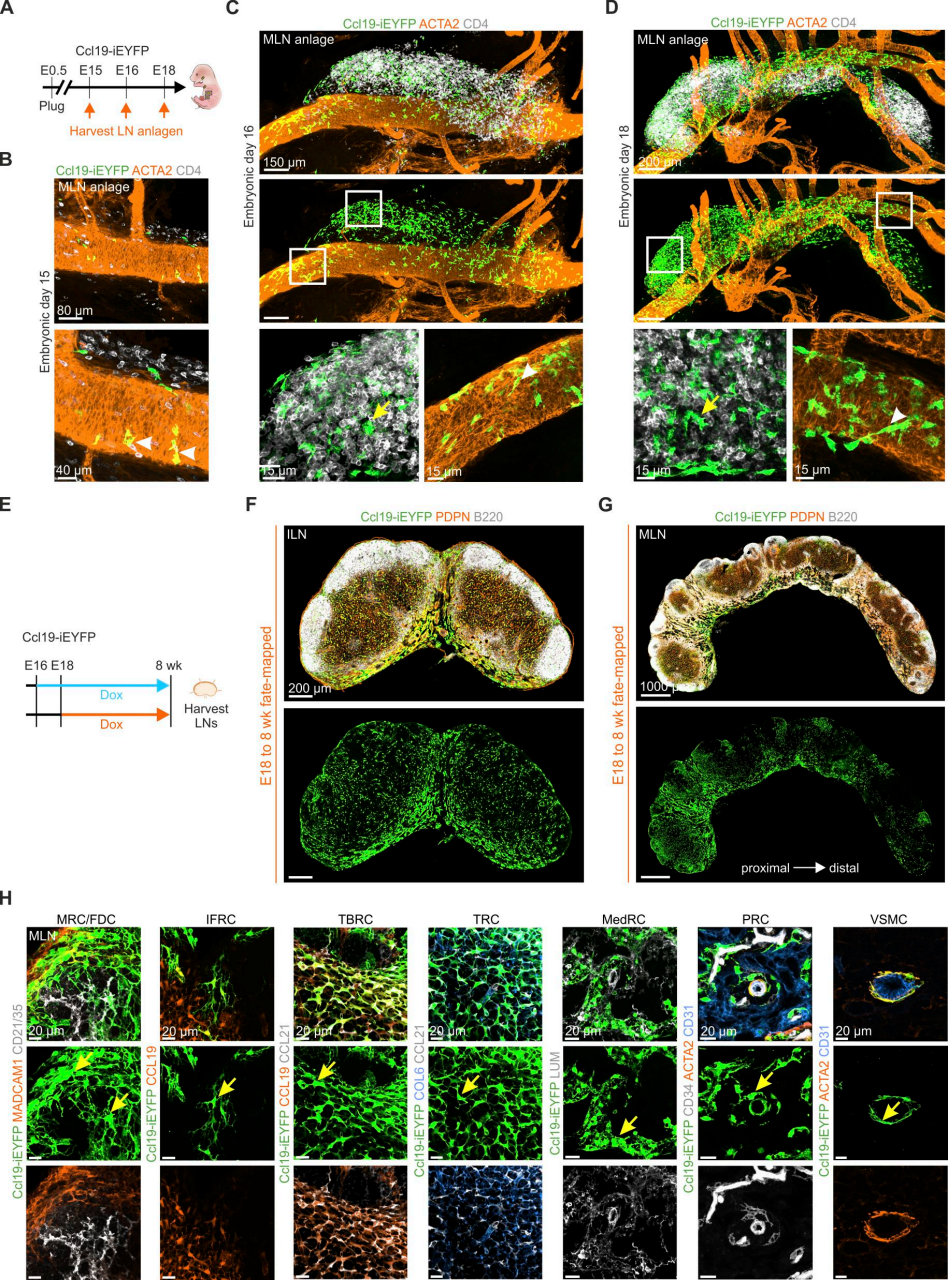
**Characterization of Ccl19-iEYFP** ^**+**^ **progenitors in lymph node anlagen and cell fate analysis in peripheral and mesenteric lymph nodes. (A)** Schematic representation for the analysis of inguinal and mesenteric lymph node anlagen from Ccl19-iEYFP embryos at the indicated time points. **(B–D)** Whole-mount confocal microscopy analysis of mesenteric lymph node anlagen from Ccl19-iEYFP^+^ embryos at E15 (B), E16 (C), and E18 (D). Boxed areas indicate regions of higher magnification. Arrows and arrowheads indicate the localization of Ccl19-tTA^+^ cells. High-resolution images are representative for three inguinal and three mesenteric lymph node anlagen from three independent experiments. Scale bars: 80 and 40 µm (B), 150 µm (C, upper panels) and 15 µm (C, lower panels), and 200 µm (D, upper panels) and 15 µm (D, lower panels). **(E)** Schematic of cell fate analysis of inguinal and mesenteric lymph nodes from Ccl19-iEYFP^+^ mice. **(F and G)** Fate-mapping analysis of EYFP^+^ cells in inguinal (F) and mesenteric (G) lymph nodes harvested from adult Ccl19-iEYFP^+^ mice after Dox administration starting at E18. Microscopy images are representative for three inguinal and three mesenteric lymph nodes from three independent experiments. Scale bars: 200 µm (F) and 1,000 µm (G). **(H)** Localization and appearance of FRC subsets and VSMCs in cross sections of mesenteric lymph nodes. High-resolution microscopy images are representative for three mesenteric lymph nodes from three independent experiments. Scale bar: 20 µm (H). Figure was complemented with elements from https://BioRender.com.

**Figure S3.**
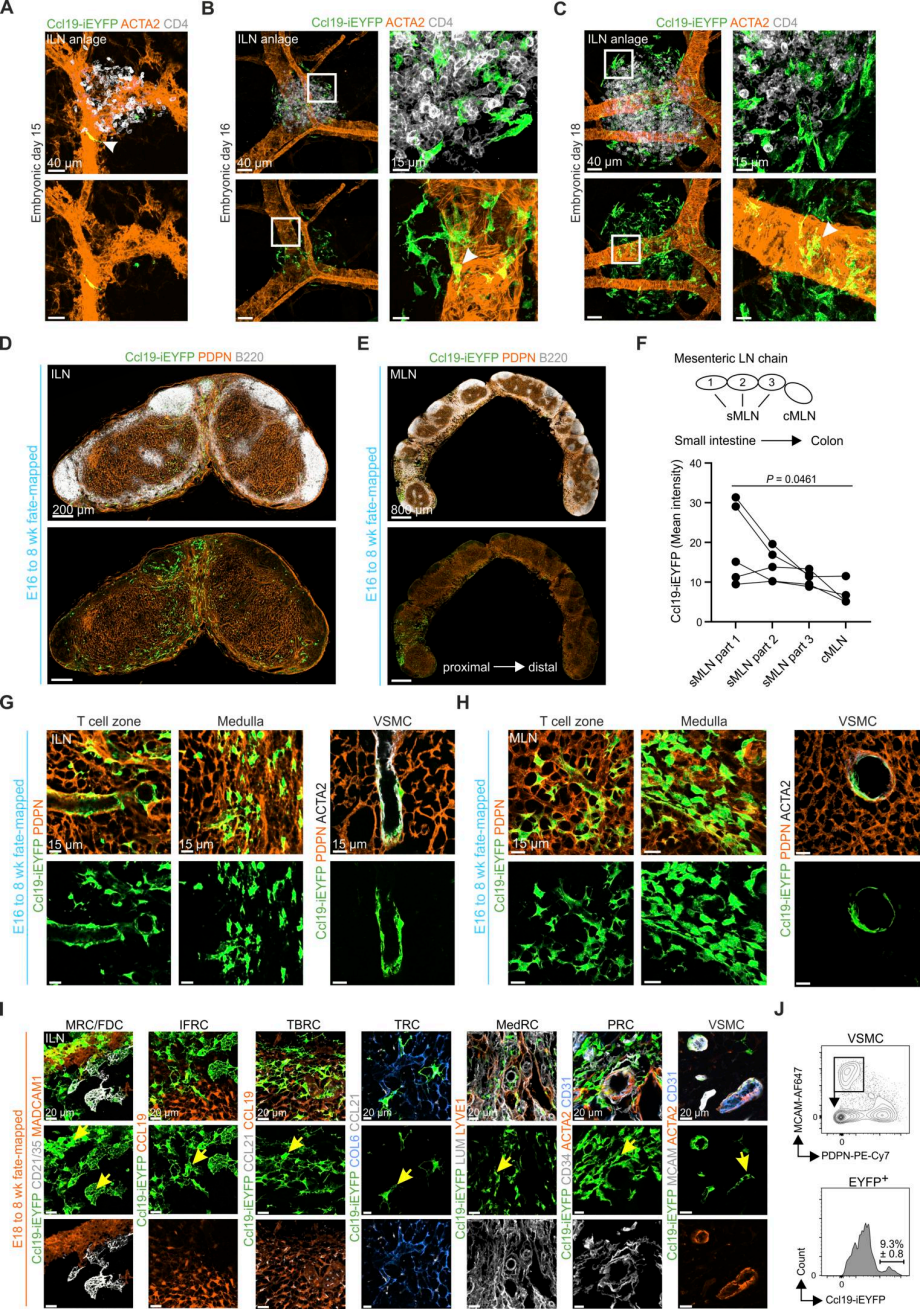
**Cell fate analysis of Ccl19-tTA** ^**+**^ **progenitors in peripheral and mesenteric lymph nodes. (A–C)** Whole-mount confocal microscopy images showing inguinal lymph node anlagen from Ccl19-iEYFP^+^ embryos at E15 (A), E16 (B), and E18 (C). Boxed areas indicate regions of higher magnification. Arrowheads indicate appearance of Ccl19-tTA^+^ LTo cells around the blood vessel. High-resolution microscopy images are representative for three inguinal lymph node anlagen from three independent experiments. Scale bars: 40 µm (A); 40 and 15 µm (B and C, left and right panels). **(D and E)** Fate-mapping analysis of EYFP^+^ cells in inguinal and mesenteric lymph nodes harvested from adult Ccl19-iEYFP^+^ mice after Dox administration starting at E16. Microscopy images are stitched tile scans and representative for three inguinal and three mesenteric lymph nodes from three independent experiments. Scale bars: 200 µm (D) and 800 µm (E). **(F)** Quantitative analysis of the Ccl19-iEYFP transgene activity along the mesenteric lymph node chain of fate-mapped mice (E18 to adult). Data are shown as mean intensity values of *n* = 5 mice from three independent experiments. Adjusted P value was calculated using the Kruskal–Wallis test for Dunn’s multiple comparisons. **(G and H)** Confocal microscopy images showing localization of EYFP^+^ cells in the T cell zone and medulla in cross sections of inguinal (G) or mesenteric (H) lymph nodes after fate-mapping from E16. High-resolution microscopy images are representative for three inguinal and three mesenteric lymph nodes from three independent experiments. Scale bars: 15 µm. **(I)** Localization and appearance of FRC subsets and VSMCs in cross sections of inguinal lymph nodes from adult Ccl19-iEYFP^+^ mice after Dox administration starting at E18. High-resolution microscopy images are representative for three inguinal lymph nodes from three independent experiments. Scale bars: 20 µm. **(J)** Flow cytometric analysis of VSMCs in mesenteric lymph nodes from adult Ccl19-iEYFP^+^ mice after Dox administration starting at E18 (*n* = 3). sMLN, small intestinal mesenteric lymph node; cMLN, colonic mesenteric lymph node.

The finding that lymph node VSMCs were lineage traced by the Ccl19-tTA and Cxcl13-Cre transgenes suggested that lymph node FRCs and VSMCs share a developmental origin. To identify a potential common progenitor cell in developing lymph nodes of Ccl19-iEYFP mice, we first used timed pregnancy and histological analysis of lymph node anlagen at different embryonic stages (Fig. 3 A). The earliest transgene activity in both inguinal and mesenteric lymph node anlagen was detected at embryonic day (E) 15 in perivascular fibroblasts surrounded by CD4^+^ lymphoid tissue inducer (LTi) cells (Fig. 3 B and Fig. S3 A, arrowheads). As described previously for Ccl19-Cre^+^ lymphoid tissue organizer (LTo) cells (Chai et al., 2013), the population of Ccl19-tTA^+^ LTo cells expanded substantially at the junction of the subepigastric veins in the inguinal fat pad (Fig. S3, B and C). Likewise, expanding Ccl19-tTA^+^ LTo cells in the mesenteric lymph node primordium were situated in proximity to the major mesenteric veins and were surrounded by CD4^+^ LTi cells (Fig. 3, C and D, arrows). A fraction of the Ccl19-tTA^+^ LTo cells in both lymph node anlagen were found in close proximity to ACTA2^+^ VSMCs (Fig. 3, C and D; and Fig. S3, B and C, arrowheads), suggesting that the commitment of CCL19-expressing LTo cells is initiated in the perivascular space.

To follow the respective FRC and mural cell differentiation trajectories, we arrested Cre-mediated recombination in early transgene-expressing LTo cells by administering Dox to the drinking water of pregnant Ccl19-iEYFP dams at E16 and E18 and harvested adult inguinal and mesenteric lymph nodes from the offspring after continuous Dox treatment (Fig. 3 E). Fate-mapped FRCs and VSMCs in adult lymph nodes (E16 to 8 wk) were found mostly in perivascular areas of the T cell zone and the medulla of inguinal (Fig. S3 D) and mesenteric lymph nodes (Fig. S3E). When Dox treatment was initiated at E18, fate-mapped cells underpinned cortical, paracortical, and medullary regions of inguinal (Fig. 3 F) and mesenteric lymph nodes (Fig. 3 G). The abundance of EYFP^+^ cells decreased from the proximal to the distal end of the mesenteric lymph node chain (Fig. 3 G and Fig. S3, E and F), which most likely reflects the proximal-to-distal developmental pattern of the gastrointestinal tract (Kolev and Kaestner, 2023). High-resolution confocal microscopy of E16–8 wk (Fig. S3, G and H) and E18–8 wk fate-mapped cells (Fig. 3 H and Fig. S3 I) confirmed that EYFP-expressing embryonic LTo cells differentiated into MADCAM1^+^ MRCs, CD21/35^+^ FDCs, CCL19^+^ IFRCs, CCL19^+^ CCL21^+^ TBRCs, COL6^+^ CCL21^+^ TRCs, LUM^+^ MedRCs, PDPN^+^ PRCs, and ACTA2^+^ VSMCs in both mesenteric (Fig. 3 H) and inguinal lymph nodes (Fig. S3 I). Approximately 10% of adult VSMCs were derived from E18 progenitors in the mesenteric lymph node anlage at E18 (Fig. S3 J). In sum, the cell fate-mapping analysis demonstrates that Ccl19-tTA fate-mapped LTo cells in the inguinal and mesenteric lymph node anlagen possess the potential to differentiate into FRCs and VSMCs.

### Molecular definition of the common FRC and VSMC progenitor

**Figure 4.**
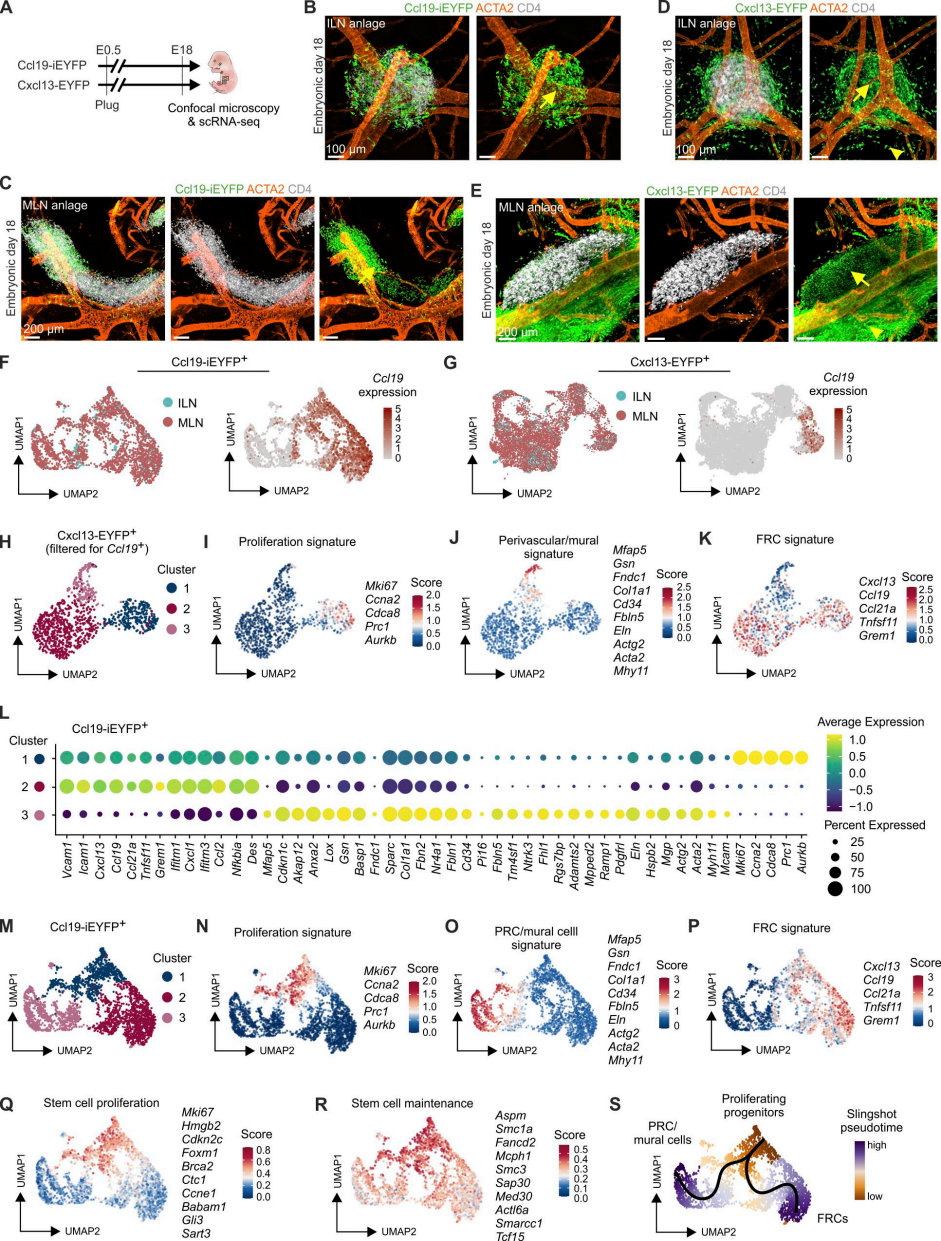
**Molecular characterization of Ccl19-tTA** ^**+**^ **and Cxcl13-Cre** ^**+**^ **FRC and VSMC progenitors in inguinal and mesenteric lymph node anlagen. (A)** Schematic representation for the analysis of inguinal and mesenteric lymph node anlagen from Ccl19-iEYFP^+^ and Cxcl13-EYFP^+^ embryos at E18. **(B and C)** Whole-mount confocal microscopy analysis of inguinal (B) and mesenteric (C) lymph node anlagen from Ccl19-iEYFP embryos at E18. Arrows highlight the localization of Ccl19-iEYFP^+^ cells inside the lymph node anlage. Microscopy images are representative for three inguinal and three mesenteric lymph node anlagen from three independent experiments. Scale bars: 100 µm (B) and 200 µm (C). **(D and E)** Whole-mount confocal microscopy images showing inguinal (D) and mesenteric (E) lymph node anlagen from Cxcl13-EYFP^+^ embryos at E18. Arrows highlight the localization of Cxcl13-EYFP^+^ cells in the lymph node anlage. Arrowhead highlights the appearance of Cxcl13^+^ cells in the mesenchyme around the lymph node anlage. Microscopy images are representative for three inguinal and three mesenteric lymph node anlagen from three independent experiments. Scale bars: 100 µm (D) and 200 µm (E). **(F)** scRNA-seq analysis of Ccl19-iEYFP^+^ cells from inguinal and mesenteric lymph node anlagen at E18. UMAP representation of Ccl19-iEYFP^+^ cell clusters colored by lymph node entity (left panel) and *Ccl19* expression (right panel). **(G)** scRNA-seq analysis of Cxcl13-EYFP^+^ cells from inguinal and mesenteric lymph node anlagen at E18. UMAP representation of Cxcl13-EYFP^+^ cell clusters colored by lymph node entity (left panel) and *Ccl19* expression (right panel). **(H)** scRNA-seq analysis of Cxcl13-EYFP^+^ cells filtered for *Ccl19* expression colored by cluster identity. **(I–K)** UMAP representation of Cxcl13-EYFP^+^*Ccl19*^+^ cells and projection of genes associated with cell proliferation (I), perivascular/mural (J), and FRC (K) signatures on the scRNA-seq dataset. **(L–S)** scRNA-seq analysis of Ccl19-iEYFP^+^ cells isolated from inguinal and mesenteric lymph node anlagen at E18. **(L)** Dot plot indicating the average expression of signature genes across embryonic Ccl19-iEYFP^+^ cell populations. **(M)** UMAP representation of Ccl19-iEYFP^+^ cell clusters. **(N–P)** Projection of proliferation (N), perivascular/mural (O), and FRC (P) signatures consisting of the indicated genes on the scRNA-seq dataset. **(Q and R)** Projection of selected genes associated with stem cell proliferation (Q) and stem cell population maintenance (R) on the scRNA-seq dataset. **(S)** Differentiation trajectory analysis of Ccl19-iEYFP^+^ cells from mesenteric lymph nodes using slingshot colored by the inferred slingshot pseudotime. Lymph node anlagen scRNA-seq data are representative of *n* = 15 embryos from two independent experiments; 2,699 cells in total from Ccl19-iEYFP^+^ embryos and 8,787 cells from Cxcl13-EYFP^+^ embryos.

**Figure S4.**
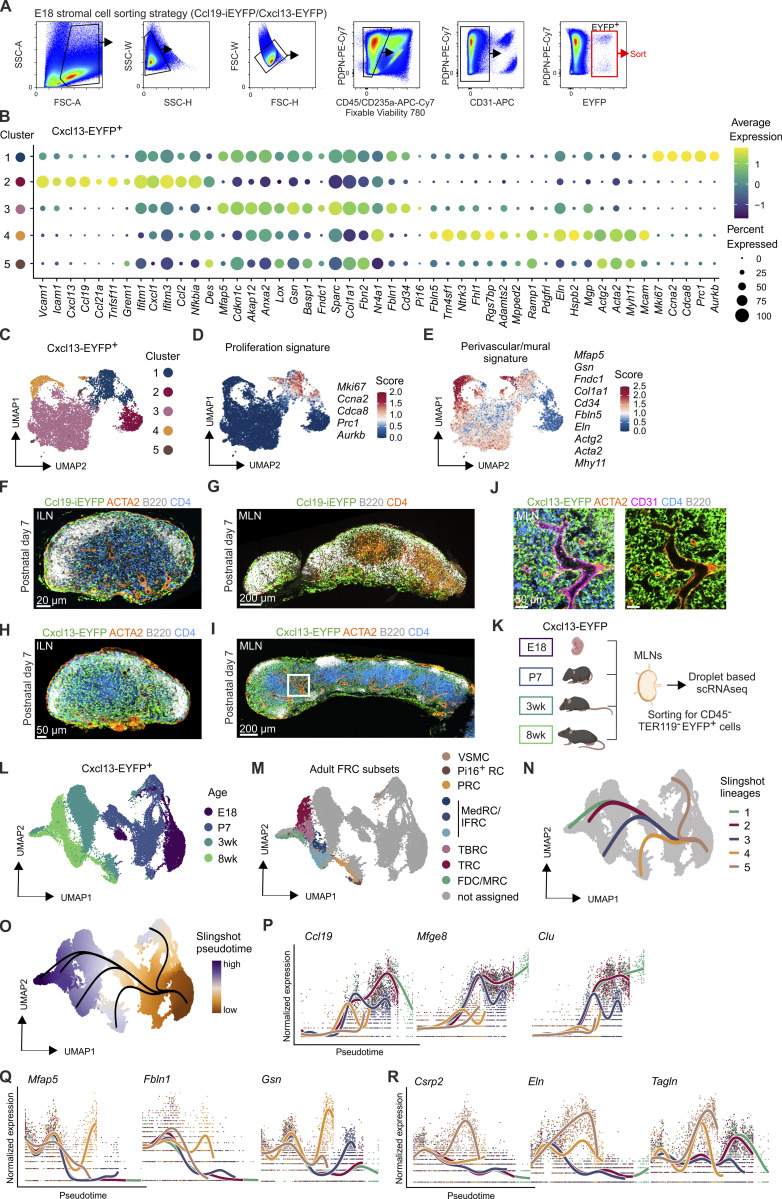
**Differentiation trajectories of Ccl19-iEYFP** ^**+**^ **and Cxcl13-EYFP** ^**+**^ **FRCs and VSMCs in murine lymph nodes. (A)** Sorting strategy of EYFP^+^ progenitors for the scRNA-seq analysis of lymph node anlagen from Ccl19-iEYFP^+^ and Cxcl13-EYFP^+^ embryos at E18. **(B)** Dot plot indicating the average expression of signature genes across embryonic Cxcl13-EYFP^+^ cell populations. **(C–E)** UMAP representation of Cxcl13-EYFP^+^ cell clusters (C) and projection of proliferation (D) and perivascular/mural (E) signatures consisting of the indicated genes on the scRNA-seq dataset. **(F and G)** Confocal microscopy images showing cross sections of inguinal (F) and mesenteric (G) lymph nodes isolated on postnatal day 7 from Ccl19-iEYFP^+^ pups. Microscopy images are representative for three inguinal and three mesenteric lymph nodes from three independent experiments. Scale bars: 20 µm (F) and 200 µm (G). **(H–J)** Confocal microscopy images showing cross sections of inguinal (H) and mesenteric (I) lymph nodes isolated on postnatal day 7 from Cxcl13-EYFP^+^ pups. Boxed areas indicate regions of higher magnification (J). Microscopy images are representative for three inguinal and three mesenteric lymph nodes from three independent experiments. Overviews in G and I are stitched images from tile scans. Scale bars: 50 µm (H), 200 µm (I), and 50 µm (J). **(K)** Schematic representation of the workflow for transcriptome analysis of EYFP^+^ cells from mesenteric lymph nodes of Cxcl13-EYFP mice using droplet-based scRNA-seq. **(L and M)** UMAP visualizing Cxcl13-Cre^+^ cells from mesenteric lymph nodes colored by (L) age group and (M) FRC subset identity derived from the collective analysis of all adult FRCs. **(N and O)** UMAP visualizing Cxcl13-Cre^+^ cells from mesenteric lymph nodes with the inferred differentiation lineages from slingshot analysis (N) and cells colored by the inferred slingshot pseudotime (O). **(P–R)** Selected differentially expressed genes in TRC/BRC/MedRC lineages (P), PRC lineage (Q), and VSMC lineage (R) along the pseudotime. Mesenteric lymph node scRNA-seq data are representative of *n* = 10−15 mice per time point; 6,029 cells from E18; 8,219 cells from P7; 7,512 cells from 3-wk-old mice; 6,509 cells from 8-wk-old mice. Lymph node anlagen scRNA-seq data are representative of *n* = 15 embryos from two independent experiments; 2,699 cells in total from Ccl19-iEYFP^+^ embryos and 8,787 cells from Cxcl13-EYFP^+^ embryos.

To define the molecular features of CCL19-expressing FRC and mural cell progenitors in the broader context of the developing tissue, we compared inguinal and mesenteric lymph node anlagen from E18 embryos of Ccl19-iEYFP and Cxcl13-EYFP mice by confocal microscopy and scRNA-seq (Fig. 4 A). While EYFP expression in both inguinal (Fig. 4 B, arrow) and mesenteric (Fig. 4 C, arrow) lymph node anlagen in Ccl19-iEYFP embryos was confined to the area covered by CD4^+^ LTi cells, the more pervasive *Cxcl13* promoter activity resulted in EYFP expression both in the LTi cell–defined lymph node anlagen (Fig. 4, D and E, arrows) and in the surrounding mesenchyme (Fig. 4, D and E, arrowheads). scRNA-seq analysis of sorted CD45^−^ Ter119^−^ CD31^−^ EYFP^+^ cells from the lymph node anlagen (Fig. S4 A) revealed that, as expected, the majority of lineage-traced EYFP^+^ cells from Ccl19-iEYFP anlagen showed current expression of *Ccl19* (Fig. 4 F), whereas only a small fraction of EYFP^+^ cells from the Cxcl13-EYFP lineage expressed *Ccl19* mRNA (Fig. 4 G). Unbiased clustering with computation of key signature genes in the Cxcl13-EYFP lineage revealed that *Ccl19* expression was confined to cluster 2, expressing other FRC genes such as *Cxcl13*, *Ccl21a*, *Tnfsf11*, and *Grem1*, and a fraction of cluster 1 (Fig. S4, B and C), with the latter being signified by the cell proliferation–associated genes, including *Mki67*, *Ccna2*, and *Cdca8* (Fig. S4, B–D). The cell proliferation signature was also found in a fraction of the Cxcl13-Cre lineage-traced cells with gene expression patterns of perivascular and mural cells (Fig. S4, D and E), highlighting potential progenitor cells inside and outside the developing lymph node. Indeed, filtering for *Ccl19*-expressing cells in the Cxcl13-Cre lineage (Fig. 4, H and I) confirmed that proliferation is a major trait of the cells in the lymph node anlage displaying the FRC gene signature (Fig. 4, I–K). In-depth analysis of EYFP^+^ cells from Ccl19-iEYFP anlagen, i.e., the cells that form the fibroblastic backbone of the developing lymph node, confirmed the presence of three phenotypically distinct clusters (Fig. 4, L and M) with the proliferation signature in cluster 1 (Fig. 4 N), perivascular and mural cell signatures in cluster 3 (Fig. 4 O), and the FRC signature genes being expressed in clusters 1 and 2 (Fig. 4 P). Increased expression of gene sets highlighting stem cell proliferation (Fig. 4 Q) and stem cell maintenance (Fig. 4 R) in proliferating, *Ccl19*-expressing cells of cluster 1 indicated the potential progenitor population of both FRCs and mural cells in the lymph node anlage. Indeed, pseudotime analysis based on the slingshot algorithm identified two distinct differentiation trajectories (Fig. 4 S). In sum, these data underscore that the dynamic microenvironment of the lymph node anlage generates both FRCs and mural cells from a common, *Ccl19*-expressing progenitor.

### Distinct transcriptional niche programs govern FRC and VSMC development

**Figure 5.**
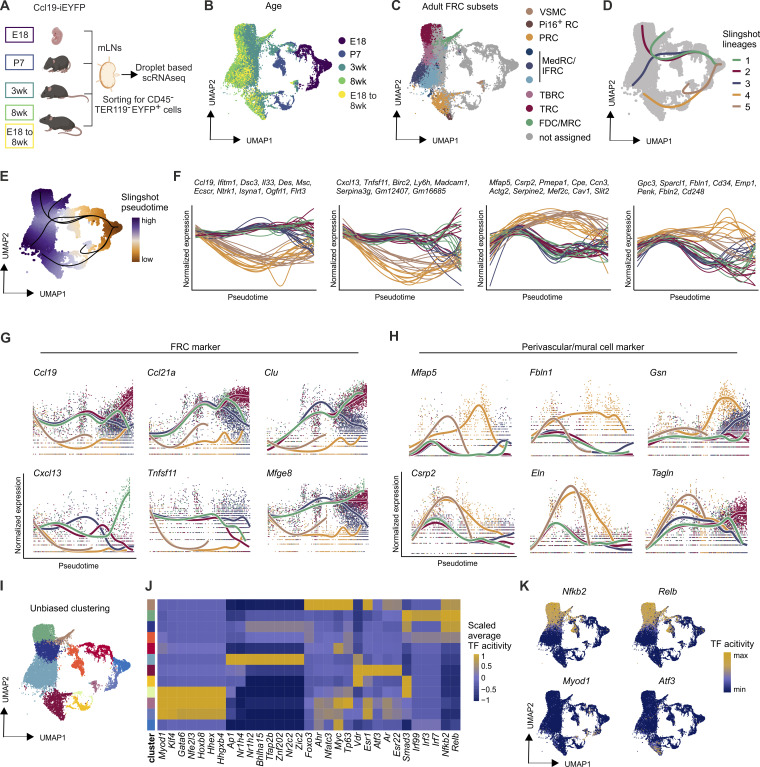
**Differentiation trajectories of FRCs and VSMCs in murine lymph nodes. (A)** Schematic representation of the workflow for transcriptome analysis of FRCs from mesenteric lymph nodes of Ccl19-iEYFP mice using droplet-based scRNA-seq. **(B and C)** UMAP visualizing Ccl19-iEYFP^+^ cells from mesenteric lymph nodes colored by (B) age group and (C) FRC subset identity derived from the collective analysis of all adult FRCs. **(D and E)** UMAP visualizing Ccl19-iEYFP^+^ cells from mesenteric lymph nodes with the inferred differentiation lineages from slingshot analysis (D) and cells colored by the inferred slingshot pseudotime (E). **(F)** Expression fits of the assigned genes along the pseudotime for each of the inferred slingshot lineages. Genes that have similar expression patterns along all lineages were clustered, and clusters with >7 genes are shown. **(G)** Selected differentially expressed genes in the slingshot TRC/BRC/MedRC lineages along the pseudotime. **(H)** Selected differentially expressed genes in the slingshot PRC/VSMC lineages along the pseudotime. **(I)** UMAP visualizing Ccl19-iEYFP^+^ cells from mesenteric lymph nodes colored by cluster identity inferred from unbiased clustering. **(J)** Heatmap showing the scaled average activity of transcription factors (TFs) across clusters of Ccl19-iEYFP^+^ cells from mesenteric lymph nodes. For each adult FRC/VSMC cluster the top five transcription factors with the highest averages activity are shown. **(K)** Scaled activity in each cell of the most active transcription factors for adult FRC/VSMC clusters projected on the UMAP of Ccl19-iEYFP^+^ cells from mesenteric lymph nodes. Mesenteric lymph node scRNA-seq data are representative of *n* = 10–15 mice per time point; 33,903 cells from E18; 10,005 cells from P7; 12,291 cells from 3-wk-old mice; 52,188 cells from 8-wk-old mice.

The developing lymph node expands during the postnatal week with the segregation of T and B cell zones, the establishment of the medullary infrastructure and further extension of the vasculature (Onder et al., 2017). Ccl19-tTA^+^ FRCs supported the compartmentalization of both inguinal and mesenteric lymph nodes at postnatal day 7 (Fig. S4, F and G). Likewise, Cxcl13-Cre^+^ cells underpinned the developing inguinal and mesenteric lymph nodes (Fig. S4, H and I) and densely populated the perivascular niche (Fig. S4 J). To fully elaborate the progenitor/progeny relationship of *Ccl19*-expressing embryonic progenitors to adult FRCs and VSMCs, including the early postnatal period, we performed time-resolved single-cell transcriptomics analysis focusing on the mesenteric lymph node (Fig. 5 A). In addition, we included Ccl19-tTA^+^ E18-8 wk fate-mapped cells (E18 to 8 wk) to the analysis (Fig. 5 A). UMAP representation showed clustering of Ccl19-tTA^+^ cells according to their developmental stage (Fig. 5 B). Projection of the cell subset identity derived from the analysis of adult mesenteric FRCs and VSMCs (Fig. 2 A) showed the transcriptional identity of 8 wk Ccl19-tTA^+^ cells and E18 to 8 wk fate-mapped cells (Fig. 5, B and C), confirming that adult lymph node FRCs and VSMCs were derived from CCL19-expressing embryonic precursors. Using the slingshot trajectory analysis, we found five differentiation trajectories from embryonic LTo cells to adult FDC/MRC, TRC, MedRC/IFRC, PRC and VSMC clusters (Fig. 5 D). The increasing slingshot pseudotime indicated early branching at E18 into two main trajectories (Fig. 5 E). Time course scRNA-seq analysis of mesenteric lymph node FRCs and mural cells expressing the Cxcl13-EYFP transgene confirmed the presence of distinct differentiation pathways starting from Cxcl13-EYFP^+^ cell populations of the mesenteric lymph node anlage to adult FRC and mural cell populations (Fig. S4, L–O). Analysis of differentially expressed genes along the pseudotime for each of the slingshot lineages demonstrated the presence of unique transcriptional programs that drive the differentiation of specialized FRC subsets when compared with the genetic programs that are associated with PRC and VSMC differentiation (Fig. 5 F). For example, we detected upregulation of immune-stimulatory genes, including *Ccl19*, *Cxcl13*, *Ccl21a*, *Tnfsf11*, *Clu*, and *Mfge8*, with characteristic expression patterns along the trajectories leading to the specialized FRC subsets (Fig. 5 G and Fig. S4 P). In contrast, the trajectories leading to PRCs and VSMCs showed differential expression of genes, including *Mfap5*, *Csrp2*, *Fbln1*, *Eln*, *Gsn*, and *Tagln* (Fig. 5 H and Fig. S4, Q and R). To further elaborate the genetic regulatory processes underlying the differentiation processes of FRCs in comparison to VSMCs and PRCs, we examined the activity of transcription factors in FRC and VSMC clusters during lymph node development. Unbiased clustering according to their inferred activity indicated the involvement of distinct transcription factors during FRC and VSMC differentiation (Fig. 5 I). Transcription factors activated through the LTβR pathway such as *Nfkb2* and *Relb* were expressed mainly in TRC, TBRC, and FDC/MRC clusters (Fig. 5, J and K). In contrast, the activity of *Myod1*, *Klf4*, *Gata6*, *Hoxb3*, and *Hhgxb4* was predicted to be involved in the early perivascular differentiation pathway, while *Atf3*, *Ar*, and *Esr1* were predicted to contribute to PRC differentiation (Fig. 5, J and K). The analysis further predicted that lymph node VSMCs exhibit a high activity of the transcription factors *Myod1* and *Klf4* (Fig. 5, J and K), which is in line with previous analyses of smooth muscle cell differentiation (Jiang and Qian, 2023). Collectively, these data indicate that partially overlapping, yet distinct transcriptional programs drive the differentiation of FRCs and VSMCs in different niche environments of murine lymph nodes.

### PRCs and VSMCs generate a LTβR-independent perivascular niche

**Figure 6.**
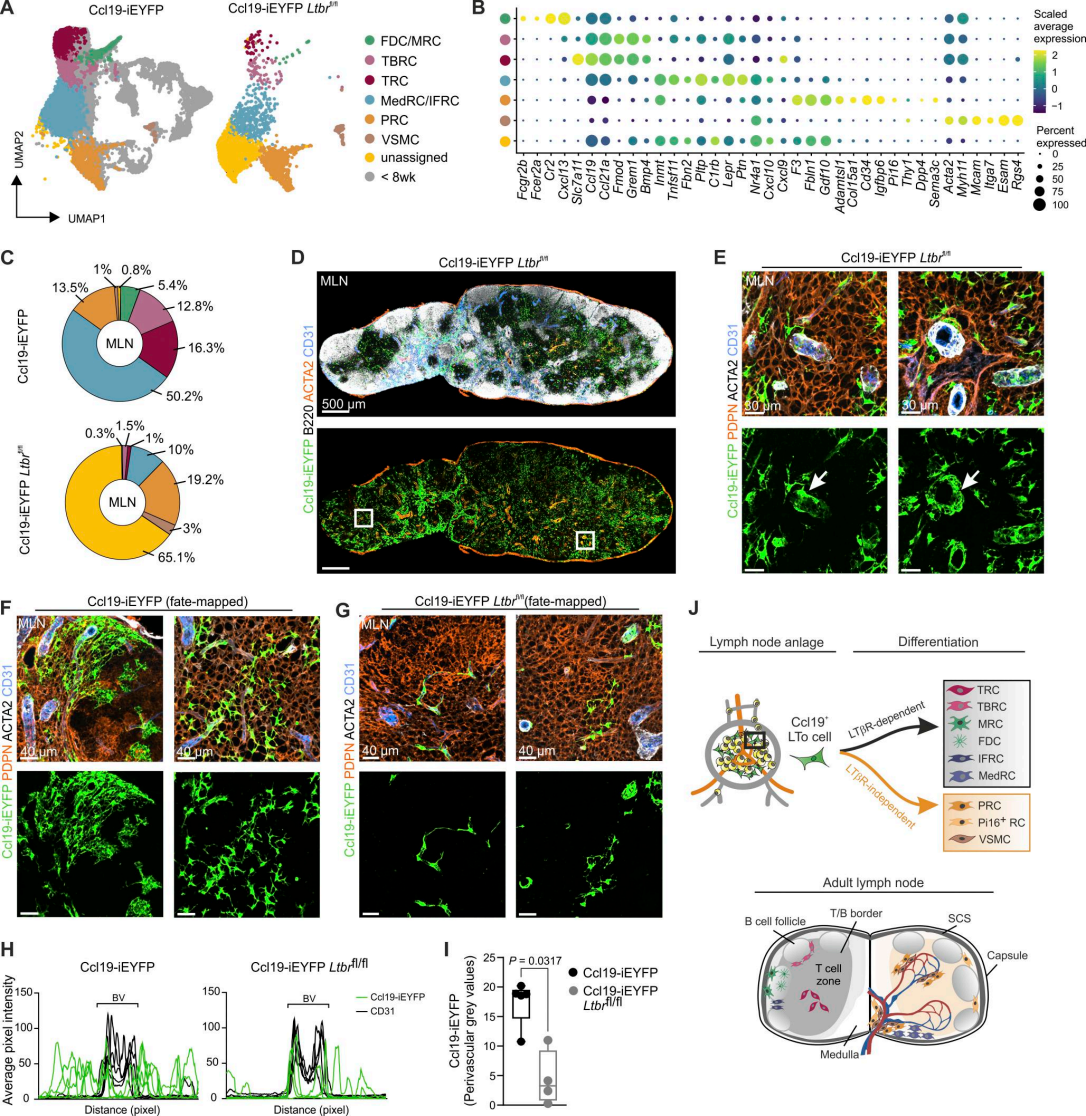
**Molecular characterization of *Ltbr*-deficient FRCs in murine lymph nodes. (A–C)** scRNA-seq data of FRCs and VSMCs from mesenteric lymph nodes of Ccl19-iEYFP and Ccl19-iEYFP *Ltbr*^fl/fl^ mice. **(A)** UMAP representation colored according to cluster identity. **(B)** Dot plot showing the average expression of signature genes in VSMCs and FRC subsets of EYFP^+^ cells isolated from mesenteric lymph nodes of Ccl19-iEYFP and Ccl19-iEYFP *Ltbr*^fl/fl^ mice. **(C)** Pie chart showing the relative abundance of FRC subsets and VSMCs in mesenteric lymph nodes from Ccl19-iEYFP and Ccl19-iEYFP *Ltbr*^fl/fl^ mice. **(D and E)** Confocal microscopy images showing cross sections of mesenteric lymph nodes from Ccl19-iEYFP *Ltbr*^fl/fl^ mice. Boxed areas indicate regions of higher magnification. Arrows indicate appearance of Ccl19-tTA^+^ cells in perivascular areas. Microscopy images are representative for three mesenteric lymph nodes from three independent experiments. Scale bars: 500 µm (D) and 30 µm (E). **(F and G)** Fate analysis of EYFP^+^ cells in mesenteric lymph nodes harvested from adult Ccl19-iEYFP^+^ (F) and Ccl19-iEYFP *Ltbr*^fl/fl^ (G) mice after Dox administration from E18. Microscopy images are representative for four mesenteric lymph nodes per condition from three independent experiments. Scale bars: 40 µm (F–G). **(H and I)** Quantification of perivascular Ccl19-iEYFP^+^ cells using histology of cross sections from mesenteric lymph nodes of fate mapped Ccl19-iEYFP and Ccl19-iEYFP *Ltbr*^fl/fl^ mice. **(H)** Average pixel intensity of Ccl19-iEYFP signal with distance from CD31^+^ blood vessels (BV). **(I)** Quantification of perivascular grey values of EYFP signal in Ccl19-iEYFP and Ccl19-iEYFP *Ltbr*^fl/fl^ mice. Data are representative of four mesenteric lymph nodes from three independent experiments (H and I). **(J)** Schematic depiction of differentiation trajectories of CCL19-expressing LTo cells in murine lymph nodes. Figure was complemented with elements from https://BioRender.com.

**Figure S5.**
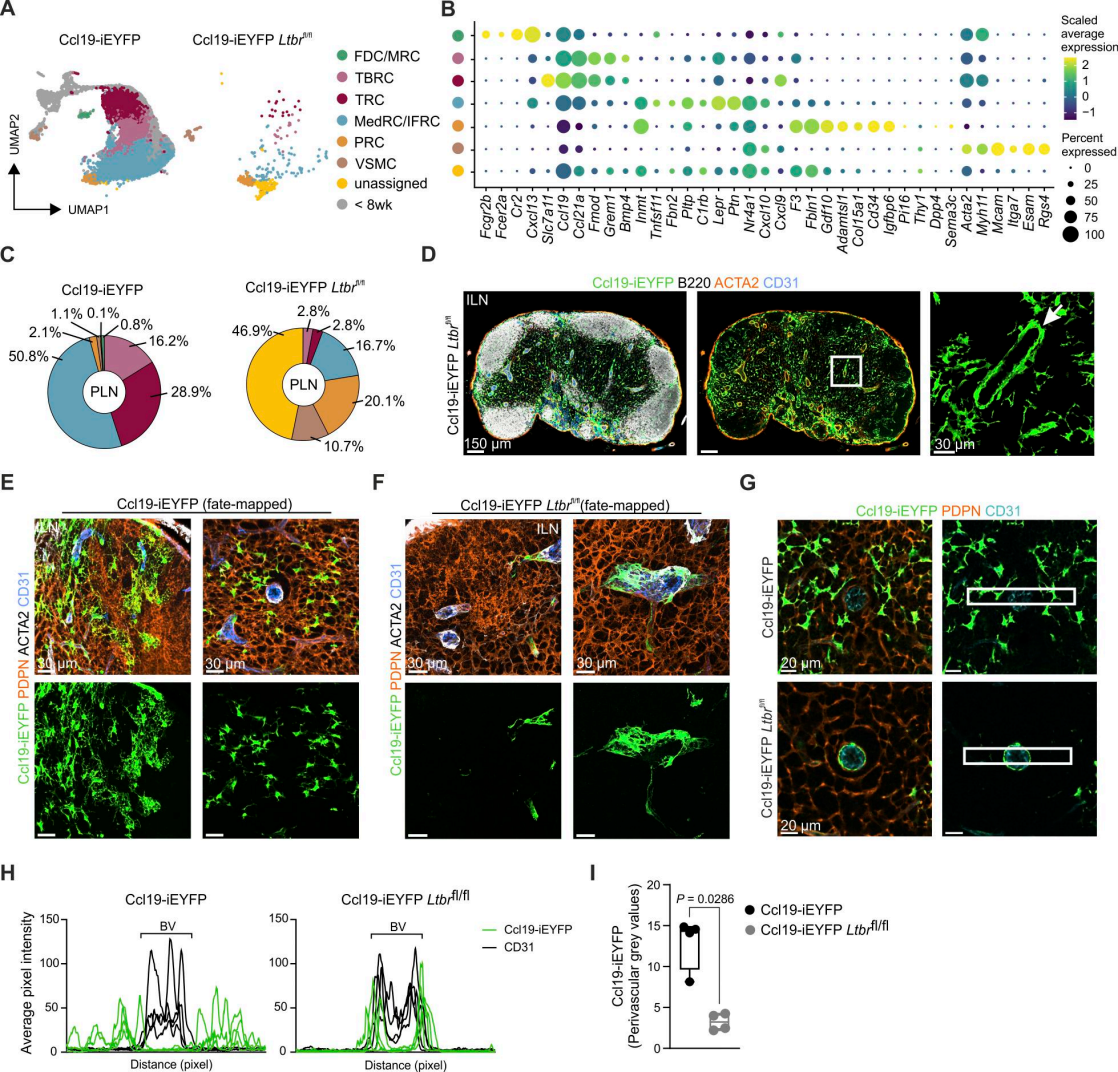
**Characterization of *Ltbr*-deficient FRCs in peripheral lymph nodes. (A–C)** scRNA-seq analysis of FRCs and VSMCs from peripheral lymph nodes of Ccl19-iEYFP and Ccl19-iEYFP *Ltbr*^fl/fl^ mice. **(A)** UMAP representation colored by cluster identity. **(B)** Dot plot showing the average expression of signature genes across VSMCs and FRC subsets of EYFP^+^ cells isolated from peripheral lymph nodes of Ccl19-iEYFP and Ccl19-iEYFP *Ltbr*^fl/fl^ mice. **(C)** Pie chart showing the relative abundance of FRC subsets and VSMCs in peripheral lymph nodes of Ccl19-iEYFP and Ccl19-iEYFP *Ltbr*^fl/fl^ mice. **(D)** Confocal microscopy images showing cross sections of inguinal lymph nodes from Ccl19-iEYFP *Ltbr*^fl/fl^ mice. Arrow indicates the localization of Ccl19-tTA^+^ cells in perivascular areas of higher magnification. Microscopy images are representative for three inguinal lymph nodes from three independent experiments. Scale bars: 150 µm (left panels) and 30 µm (right panel). **(E and F)** Fate analysis of EYFP^+^ cells in inguinal lymph nodes harvested from adult Ccl19-iEYFP^+^ (E) and Ccl19-iEYFP *Ltbr*^fl/fl^ (F) mice after Dox administration from E18. High-resolution microscopy images are representative for three inguinal lymph nodes per condition from three independent experiments. Scale bars: 30 µm. **(G–I)** Quantification of perivascular Ccl19-iEYFP^+^ cells using histology of cross sections from inguinal lymph nodes of fate-mapped Ccl19-iEYFP and Ccl19-iEYFP *Ltbr*^fl/fl^ mice (G). Scale bars: 20 µm. **(H)** Average pixel intensity of Ccl19-iEYFP signal with distance from CD31^+^ blood vessels (BV). **(I)** Quantification of perivascular grey values of EYFP signal in Ccl19-iEYFP and Ccl19-iEYFP *Ltbr*^fl/fl^ mice. Data are representative of four inguinal lymph nodes from three independent experiments (H and I).

Genetic ablation of *Ltbr* expression in *Ccl19*-expressing FRC progenitors abrogates the development of the splenic white pulp (Cheng et al., 2019) and blocks FRC maturation in lymph nodes and Peyer’s patches (Chai et al., 2013; Prados et al., 2021). To elaborate the molecular pathways and cellular differentiation trajectories depending on LTβR signaling in murine lymph nodes, we deleted *Ltbr* expression in Ccl19-tTA^+^ cells and performed scRNA-seq analysis of lineage-traced *Ltbr*-deficient cells from mesenteric (Fig. 6, A and B) and peripheral lymph nodes (Fig. S5, A and B) of Ccl19-iEYFP *Ltbr*^fl/fl^ mice. The lack of *Ltbr* expression resulted in near complete loss of FDCs, MRCs, TRCs, and TBRCs and a substantial reduction of MedRC and IFRC abundance in mesenteric and peripheral lymph nodes (Fig. 6 C and Fig. S5 C). An additional cluster of cells that showed an overall reduction of FRC signature genes such as *Cxcl13*, *Ccl21*, or *Grem1* emerged under the condition of *Ltbr* deficiency in Ccl19-tTA^+^ cells (Fig. 6 B and Fig. S5 B), highlighting the importance of LTβR signaling for the acquisition of FRC functions in the main immune cell niches. The finding that the proportion of PRCs (which included Pi16^+^ RCs at the resolution used here) and VSMCs were increased in lymph nodes of Ccl19-iEYFP *Ltbr*^fl/fl^ mice (Fig. 6 C and Fig. S5 C) indicated that the development of perivascular niche cells was not affected by the *Ltbr* deficiency. Confocal microscopy analysis of peripheral and mesenteric lymph nodes harvested from adult Ccl19-iEYFP *Ltbr*^fl/fl^ mice confirmed that the majority of Ccl19-tTA^+^ cells were located in perivascular areas (Fig. 6, D and E; and Fig. S5 D, arrows). Comparison of E18 to 8 wk fate-mapped *Ltbr*-proficient and -deficient lymph nodes by confocal microscopy revealed that *Ltbr*-proficient cells were distributed throughout the lymph node parenchyma (Fig. 6 F and Fig. S5 E), whereas *Ltbr*-deficient cells were found around or in close proximity to blood vessels in inguinal and mesenteric lymph nodes (Fig. 6, G–I; and Fig. S5, F–I). In sum, these data show that the development of perivascular niche cells in murine lymph nodes is independent of LTβR signaling and highlight the importance of lymphotoxin as a key niche factor for the differentiation of specialized FRC subsets (Fig. 6 J).

## Discussion

The present analysis provides a high-resolution analysis of the ontogeny of fibroblasts, i.e., FRCs, and mural cells in murine lymph nodes. The use of the inducible Ccl19-tTA model facilitated the definition of committed FRC progenitors in embryonic lymph nodes that retain the ability to differentiate into VSMCs. The LTβR-dependent expansion of perivenular Ccl19-tTA^+^ progenitors and their differentiation into specialized FRC subsets, i.e., MRCs/FDCs, TRCs, TBRCs, and MedRCs, highlights the critical involvement of lymphoid cells in the postcommitment process (Onder and Ludewig, 2018). The finding that the differentiation of the common progenitor toward specialized FRCs is linked to the aggregation of lymphotoxin-expressing LTi cells in the confined space of the lymph node anlage mirrors the LTi cell–dependent formation of Peyer’s patches (Eberl et al., 2004) and structural organization of the splenic white pulp by B cell–derived lymphotoxin (DeTogni et al., 1994; Gonzalez et al., 1998). The genetic perturbation analysis with *Ltbr* ablation in Ccl19-tTA^+^ cells demonstrated the close developmental relationship between VSMCs and PRCs, which resembles the LTβR-independent formation of PRCs and mural cells in the perivascular compartment of the splenic white pulp (Cheng et al., 2019). The present analysis thus identifies an overarching principle that governs the development of mural cells and FRC subsets in the lymph node environment: In the absence of LTβR signaling or with minimal LTβR signaling, the initial commitment of the perivenular progenitor drives the generation of the PRC-mural cell network by default. In contrast, the differentiation of specialized FRC networks depends on their interaction with lymphoid cells that provide lymphotoxin as the primary factor driving the differentiation of lymphocyte niches.

Fibroblasts and mural cells share a broad range of functions for the establishment of specific niche environments, including the provision of physical and molecular cues, such as specific ECM components, surface receptors, and soluble growth and differentiation factors (Muhl et al., 2020; Plikus et al., 2021). It has been suggested that fibroblasts and mural cells are derived from a common progenitor in the mesodermal lineage (Lendahl et al., 2022), a view that is supported by lineage-tracing analysis in lung development (Narvaez Del Pilar et al., 2022; Zhang et al., 2013). Previous lineage tracing of cells in mouse lymph nodes showed that mesenchymal progenitors in the lateral plate mesoderm expressing the transcription factor homeobox 6 at E8.5 develop into FRCs and blood endothelial cells (Lenti et al., 2022). Similarly, mesenchymal cells that express the odd-skipped–related transcription factor 1 in E12.5 lymph nodes exhibited multipotent developmental potential, demonstrating the capacity for differentiation into FRCs, lymphatic endothelial cells, and blood endothelial cells (Vallecillo-García et al., 2023). It appears that the expression of the type VI intermediate filament Nestin is another trait of such multipotent progenitors in murine lymph node anlagen (Koning et al., 2016). The differentiation trajectory toward specialized FRC subsets is most likely programmed around E15 with the expression of the fibroblast-activating protein (FAP) (Denton et al., 2019) and CCL19, as demonstrated in the present study. We consider it likely that FAP-expressing FRC progenitors can differentiate into PRCs and VSMCs because *FAP* is expressed in the perivascular space of human lymph nodes and upregulated during inflammation (Lütge et al., 2025). It is noteworthy that the perivascular compartment of human lymph nodes is organized similarly to that observed in mice, displaying concentric layers of VSMCs that physically connect to PRCs and PI16^+^ RCs, and sharing perivascular niche signatures in their transcriptome (Lütge et al., 2025). In sum, these data support the view that endothelial and fibroblastic cell lineages separate early during lymph node development and that the ontogeny of specialized FRCs and perivascular niche cells, i.e., VSMCs, PRCs, and Pi16^+^ RCs, is closely linked.

The delineation of the progenitor-progeny relationship during prenatal and postnatal development depends on the availability of appropriate lineage and cell fate-mapping models (Lee et al., 2022; VanHorn and Morris, 2021). The Ccl19-tTA model enables the reconstruction of the differentiation trajectory of progenies originating from proximal FRC progenitors that are present in the embryonic lymph node anlage (Onder et al., 2017). Unlike fate-mapping approaches that use early mesenchymal cell markers (Lenti et al., 2022; Vallecillo-García et al., 2023), utilization of the *Ccl19* promoter avoids the broad labeling of cell types other than the lymphoid organ fibroblast lineage. Accordingly, it is conceivable that FRCs in adult lymph nodes can originate from *Ccl19*-negative mLTo cells at a stage preceding *Ccl19* expression or that a fraction of FRCs develop along a differentiation trajectory toward *Ccl19*-negative FRCs. To address this potential limitation of the *Ccl19* promoter-based models, we utilized the Cxcl13-Cre–dependent FRC lineage tracing to enable tracking of *Cxcl13*^+^ BRCs (Pikor et al., 2020), which are underrepresented in the Ccl19-Cre and Ccl19-tTA approaches. Combined analysis of lineage-traced and fate-mapped FRCs from *Cxcl13*^+^*Ccl19*^+^ E18 progenitors supports the conclusion that FRCs and VSMCs can differentiate from proximal *Ccl19*-expressing progenitors in the lymph node anlage at E18.

Mural cells form the basement membrane of blood vessels in all organs and generate the vascular tone depending on their location along the vascular tree (Hartmann et al., 2022; Muhl et al., 2020; Muhl et al., 2022). The distinction of pericytes and VSMCs in lymph nodes is challenging because commonly used pericyte markers, such as the neuron-glial antigen 2 (NG2, gene name *CSPG4*), is expressed in human lymph nodes by pericytes in the walls of larger vessels, whereas pericytes around HEVs and other small vessels are negative for NG2 (Eom et al., 2020). *CSPG4* expression could be detected in a subset of *ACTA2*-expressing PRCs in human tonsils (De Martin et al., 2023) and is broadly expressed in VSMCs of human lymph nodes (Lütge et al., 2025). Likewise, classification of lymphoid organ pericytes by the expression of the alpha integrin-7 (Malhotra et al., 2012) is challenging because this adhesion molecule is expressed by human tonsillar PRCs (De Martin et al., 2023), human lymph node PRCs (Lütge et al., 2025), and murine VSMCs and PRCs, as shown by the data presented in this study. Our analysis further indicates that pericyte markers such as *TBX18* or *SCL1A3* (Plikus et al., 2021) are not exclusively expressed by mural cells in murine lymph nodes. In contrast, the transcriptional phenotype of VSMCs can be reliably assigned, and their phenotypical identity and location can be validated by confocal microscopy and flow cytometry in different human lymphoid organs (De Martin et al., 2023; Lütge et al., 2025). Since FRCs and VSMCs share common CCL19-expressing progenitors in murine lymph nodes and due to the close lineage relationship between pericytes and fibroblasts, it is reasonable to assume that pericytes develop along the same trajectory as PRCs and VSMCs. Future studies using the power of high-resolution spatial transcriptomics (Pentimalli et al., 2024) and further development of in vitro differentiation models (Sitnik et al., 2016) will be most helpful to fully resolve cell type and subset identity in the perivascular space in human and murine lymph nodes.

In conclusion, this study elucidates the ontogeny of lymph node FRCs and VSMCs from their committed progenitors and demonstrates the close lineage relationship between PRCs and mural cells in the perivascular niche environment.

## Materials and methods

### Mice

The generation of the triple-transgenic Ccl19-iEYFP mice was described previously (Cheng et al., 2019). C57BL/6N-Tg(Ccl19-tTA)^688BIAT^(Ccl19-tTA) were crossed to 26Sor^tm1(EYFP)Cos^/J (R26R-EYFP) and LC-1 mice to obtain Ccl19-iEYFP mice. The LC-1 strain (Schonig et al., 2002) was kindly provided by Dr. Fendler, Max Delbrück Center of Molecular Medicine, Berlin, Germany. Ccl19-iEYFP mice were crossed to Ltbr^tm1.1Thhe^ (*Ltbr*^fl/fl^) (Wimmer et al., 2012) to obtain conditional deletion of *Ltbr* in Ccl19-tTA^+^ cells. The generation of BAC-transgenic C57BL/6N-Tg (Ccl19-Cre)^489Biat^(Ccl19-Cre) mice and C57BL/6N-Tg(Cxcl13-Cre)^723Biat^× B6.129 × 26Sor^tm1(EYFP)Cos^/J (R26R-EYFP) (Cxcl13-Cre/TdTomato EYFP) has been described previously (Chai et al., 2013; Onder et al., 2017). All mouse strains were on a C57BL/6NCrl genetic background and housed in individually ventilated cages under specific pathogen–free conditions. A 12-h light–dark cycle, 22°C ambient temperature and 45–50% humidity was maintained. The experiments were conducted using 6–12-wk-old mice (males and females) in randomized experimental groups and in accordance with Swiss federal and cantonal guidelines (Tierschutzgesetz) under the permission numbers SG/04/2022, SG/08/2021, SG/15/2023, and SG/02/2024 granted by the Veterinary Office of the Canton of St. Gallen. For cell fate-mapping experiments, pregnant dams were treated with Dox (Sigma-Aldrich) in the drinking water (1 mg/ml).

### Preparation of lymph node stromal cells

For stromal cell isolation from peripheral (inguinal, brachial and axillary) and mesenteric lymph nodes, tissues were disrupted into small pieces using small needles and collected in RPMI 1640 medium containing 2% FCS, 20 mM HEPES, pH 7.2 (Lonza), 0.2 mg/ml collagenase P, Dispase 30 μg/ml (Roche), and 10 μg/ml DNase I. The dissociated tissue was incubated for 45 min at 37°C; during the incubation time, the tissue was resuspended, and supernatant was collected every 15 min. Enrichment of stromal cells was performed by incubating the cell suspension with MACS anti-CD45 and anti-TER119 microbeads (Miltenyi Biotec) and passing it through MACS LS columns (Miltenyi Biotec). Single-cell suspensions were stained for further flow cytometric analysis or cell sorting.

### Flow cytometry

Cell suspensions from peripheral or mesenteric lymph nodes were incubated for 30 min at 4°C in PBS containing 1% FCS and 5 mM EDTA with the following antibodies (Table S1): anti-mouse PDPN, anti-mouse CD31, anti-mouse SCA1, anti-mouse CD45, anti-mouse Ter119, anti-mouse MCAM (all from BioLegend), anti-mouse CD157, anti-mouse CD45, anti-mouse VCAM1, anti-mouse ICAM1, anti-mouse CD34, anti-mouse LY6C, anti-mouse CD21/CD35, and anti-mouse MADCAM1 (all from BD Biosciences). LIVE/DEAD cell discrimination was performed by using either a fixable BV510 Dead Cell Staining Kit (Molecular Probes) or a fixable viability dye eFluor780 (Invitrogen) prior to antibody staining. Cells were acquired with a FACS Symphony (BD Biosciences) and analyzed with the FlowJo (v.10) software (FlowJo LLC) according to established guidelines. Cell sorting was performed using a BD FACSMelody Cell Sorter and the FACSChorus (v.1.3) software (BD Biosciences).

### Immunofluorescence confocal microscopy

Inguinal and mesenteric lymph nodes were harvested and fixed for 12–24 h at 4°C in freshly prepared 4% paraformaldehyde (Merck Millipore) under agitation. Tissues were washed in PBS containing 2% FCS and 0.1% Triton-X (Sigma-Aldrich), and the remaining fat pieces were removed using small forceps. Lymph node tissues were embedded in 4% low-melting agarose (VWR International) and sectioned into 40-μm thick sections using a vibratome (Leica VT-1200). Tissue sections were blocked in PBS containing 10% FCS, 1 mg/ml anti-Fcγ receptor (BD Biosciences), and 0.1% Triton X-100 (Sigma-Aldrich) and stained overnight at 4°C with the indicated antibodies (Table S1) in PBS containing 2% FCS and 0.1% Triton X-100 (Sigma-Aldrich). Embryonic lymph node anlagen were dissected from embryos, which were previously fixed for 24 h at 4°C with 4% paraformaldehyde (Merck Millipore) under agitation. Whole lymph node anlagen were blocked and stained overnight as indicated above. Unconjugated and biotinylated antibodies were stained with the indicated secondary antibodies or streptavidin conjugates (Table S1). Confocal microscopy was performed using the confocal microscope LSM-980 (Carl Zeiss), and images were recorded and processed with ZEN 2010 software (Carl Zeiss). Imaris Version 9.2.1 was used for image analysis. ImageJ was used to quantify Ccl19-iEYFP^+^ cells in the perivascular area of cross sections of inguinal and mesenteric lymph nodes. The perivascular grey values of the Ccl19-iEYFP signal were analyzed in distance to CD31^+^ blood vessels.

### Stromal cell sorting and scRNA-seq

CD45^−^ TER119^−^ CD31^−^ stromal cells from peripheral and mesenteric lymph nodes of Ccl19-iEYFP and Cxcl13-EYFP mice were collected and sorted as described (Fig. S2, G–I) using a BD FACSMelody cell sorter (BD Biosciences). For the analysis of adult FRCs and VSMCs from peripheral and mesenteric lymph nodes, sorted cell fractions were enriched for EYFP^+^ cells (Fig. S2, H and I) and filled up with EYFP^–^ cells up to 25,000 cells per sample. After preparing single-cell suspensions using the 10x Chromium droplet-based system (10x Genomics), cDNA libraries were generated according to protocols for the Chromium Single Cell 3′ Reagent Kit (v3 or NextGEM Chemistry). The sequencing was performed on an Illumina NovaSeq 6000 at the Functional Genomic Center Zurich. Gene expression was estimated from the sequencing output using CellRanger (v5.0.1 for Ccl19-iEYFP and v.8.0.1 for Cxcl13-EYFP data) (Zheng et al., 2017) with the Ensembl GRCm38.9 (for Ccl19-iEYFP) and GRCm38.102 (for Cxcl13-EYFP) release as a reference. Subsequent quality control included the removal of cells with very high or low UMI counts (>2.5 median absolute deviation from the median of all cells), very high or low total number of detected genes (>2.5 median absolute deviation from the median of all cells), and elevated mitochondrial gene content (>2.5 median absolute deviations above the median of all cells) and was performed in R v.4.2.1 using the R/Bioconductor package scater (v.1.24.0). Additionally, cells expressing markers for B cells, T cells, or neurons (*Ptprc*, *Cd3e*, *Cd79a*, and *L1cam*) were excluded before further analysis. The Ccl19-iEYFP and Ccl19-iEYFP *Ltbr*^fl/fl^ dataset contained 108,387 cells (61,640 cells from mesenteric lymph nodes and 46,747 cells from peripheral lymph nodes) across four different age groups (33,903 cells from E18; 10,005 cells from P7; 12,291 cells from 3-wk-old mice; 52,188 cells from 8-wk-old mice) and with 87,566 cells from Ccl19-iEYFP mice and 20,821 cells from Ccl19-iEYFP *Ltbr*^fl/fl^ mice. Downstream analysis was conducted utilizing functions implemented in the Seurat R package (v.4.3.0 and v.4.4.0) (Butler et al., 2018; Hao et al., 2021), comprising procedures such as data normalization and integration for collective assignment of adult FRC subsets across peripheral and mesenteric lymph nodes, data scaling, dimensionality reduction through PCA and UMAP, graph-based clustering, and the detection of unbiased cluster markers. Clusters from lymph nodes of adult mice were assigned according to their expression profiles of both unbiased cluster markers and canonical marker genes described in earlier studies (Lütge et al., 2023; Perez-Shibayama et al., 2020; Rodda et al., 2018). Differentially expressed genes between lymph node entities and conditions were further calculated using the FindConservedMarkers and FindMarkers functions as implemented in the Seurat R package (v.4.3.0 and v.4.4.0), and significantly enriched GO terms were determined using the enrichGO function from the clusterProfiler R/Bioconductor package (v.4.4.4).

### Inference of differentiation trajectories and transcription factor activities

Continuous differentiation pathways of Ccl19-iEYFP^+^ cells from mesenteric lymph nodes were modeled using the slingshot R package (v.2.8.0) (Street et al., 2018). For trajectory inference, adult FRC clusters from 8-wk-old mice were set as end points, whereas the cluster with cells from lymph node anlagen at E18 was used as starting point. Next, the fitGAM function of the tradeSeq R/Bioconductor package (v.1.14.0) (Van den Berge et al., 2020) was used to model the expression of the 2,000 most variable genes along the inferred differentiation lineages. Based on the fitted generalized additive models (GAMs), genes that have similar expression patterns along all lineages were clustered using the clusterExpressionPatterns function. Finally, the average activity of transcription factors in cluster of Ccl19-iEYFP^+^ cells from mesenteric lymph nodes was inferred using AUCell (Aibar et al., 2017) by running the run_aucell function as implemented in the decoupleR R package (v.2.6.0) (Badia et al., 2022).

### Online supplemental material

Fig. S1 shows the transgene activity in peripheral and mesenteric lymph nodes of Ccl19-iEYFP and Cxcl13-EYFP mice. Fig. S2 shows flow cytometric and single-cell transcriptomic analyses of FRCs and VSMCs in murine peripheral and mesenteric lymph nodes. Fig. S3 cell fate-mapping analysis of Ccl19-tTA^+^ progenitors in peripheral and mesenteric lymph nodes. Fig. S4 shows the differentiation trajectories of Ccl19-iEYFP^+^ and Cxcl13-EYFP^+^ FRCs and VSMCs in murine lymph nodes. Fig. S5 shows the characterization of *Ltbr*-deficient FRCs in peripheral lymph nodes. Table S1 provides the list of antibodies used in this study.

## Supplementary Material

Table S1 shows the antibodies used in this study.

## Data Availability

The scRNA-seq data generated in this study have been deposited in the BioStudies database (https://www.ebi.ac.uk/biostudies/) and are available under the accession codes E-MTAB-13891 and E-MTAB-15366. Processed data files can be downloaded from the figshare platform (https://figshare.com) at https://doi.org/10.6084/m9.figshare.29558621. Code used for data analysis in this project is available at GitHub (for Ccl19-iEYFP scRNA-seq analysis: https://mluetge.github.io/LNdevMouse24/; for Cxcl13-EYFP scRNA-seq analysis: https://ludewig-lab.github.io/LNdevMouse24.2).
