# Supplementary material for: A common progenitor gives rise to fibroblastic reticular cells and vascular smooth muscle cells in murine lymph nodes
Source: J Exp Med. 2025 Nov 26;223(2):e20242300. doi: 10.1084/jem.20242300 (PMC12651234; doi:10.1084/jem.20242300)
Supplement: Table S1 — shows the antibodies used in this study. [file jem_20242300_tables1.docx]

**Table S1. Antibodies used in this study.**

| Anti-mouse Ly6C PerCP-Cy5.5 (BD Biosciences, Cat#: 560525, clone: AL-21, 1:200) |
| --- |
| Anti-mouse Ter119 APCCy7 (Biolegend, Cat#: 116223, clone: TER-119, 1:200) |
| Anti-mouse CD21/35 BUV395 (BD Biosciences, Cat#: 752912, clone: 7E9, 1:200) |
| Anti-mouse MCAM AF647 (Biolegend, Cat#: 134718, clone: ME-9F1, 1:300) |
| Anti-mouse CD157 BV786 (BD Biosciences, Cat#: 741012, clone: BP-3, 1:200) |
| Anti-mouse LY6A/E BV510 (Biolegend, Cat#: 108129, clone: D7, 1:400) |
| Anti-mouse CD54 (ICAM-1) BV421 (BD Biosciences, Cat#: 564704, clone: 3E2, 1:200) |
| Anti-mouse CD106 (VCAM-1) BV711 (BD Biosciences, Cat#: 740675: clone: 429, 1:400) |
| Anti-mouse CD34 BV605 (BD Biosciences, Cat#: 750918, clone: RAM34, 1:200)  Anti-mouse CD34 eFluor660 (eBioscience, Cat#: 50-0341-82, clone: RAM34, 1:100) |
| Anti-mouse CD31 BUV805 (BD Biosciences, Cat#:741939, clone: MEC 13.3, 1:200) |
| Anti-mouse MADCAM1 BUV615 (BD Biosciences, Cat#: 751510, clone: MECA-367, 1:200) |
| Anti-mouse CD45 BV496 (BD Biosciences, Cat#: 749889. clone: 30-F11, 1:200) |
| Anti-mouse PDPN Pe-Cy7 (Biolegend, Cat#: 127412, clone: 36899, 1:800) |
| Anti-mouse CD157 APC (Biolegend, Cat#: 140208, clone: BP-3, 1:100)  Anti-mouse CD45 APC-Cy7 (Biolegend, Cat#: 103116, clone: 30-F11, 1:100)  Anti-mouse PDPN (BioLegend, Cat#: 127402, clone: 8.1.1, 1:300)  Anti-human/mouse B220 eFluor450 (Thermo Scientific, Cat#: 48045282, clone: RA3-6B2, 1:200)  Anti-mouse CD31 AF594 (Biolegend, Cat#: 102520, clone: MEC13.3, 1:400)  Anti-human/mouse aSMa Cy3 (Sigma, Clone:1A4, Cat#: C6198, 1:1000)  Anti-mouse CD4 Biotin (Biolegend, Cat#: 100508, clone: RM4-5, 1:300)  Anti-human/mouse MYH11 (abcam, Clone: SP314, Cat#: ab224804, 1:300)  Anti-human/mouse aSMa eFluor660 (eBioscience, Clone: 1A4, Cat#: 50-9760-82, 1:1000)  Anti-GFP (Takara Bio Clontech, Cat#: 632592, 1:1000)  Anti-GFP (Aves Labs Inc., Cat#: GFP-1020, 1:1000)  Anti-DsRed (Takara Bio Clontech, Cat#: 632496, 1:1000)  Anti-mouse Lyve1 eFluor660 (Thermo Scientific, Cat#: 50-0443-82, clone: ALY-7, 1:200)  Anti-mouse CD31 AlexaFluor647 (BioLegend, Cat#: 102516, clone: MEC13.3, 1:200)  Anti-mouse PNAd Biotin (BioLegend, Cat#: 120804, clone: MECA-79, 1:200)  Anti-mouse CCL21 (R&D Systems, Cat#: BAF457, 1:100)  Anti-mouse MAdCAM1 Biotin (BioLegend, Cat#: 120706, clone: MECA-367, 1:200)  Anti-mouse CD21/35 Pacific Blue (BioLegend, Cat#: 123414, clone: 7E9, 1:200)  Anti-mouse Fibroblast/COL6 (Invitrogen, Cat#: MA1-40076, clone: ER-TR7, 1:500)  Anti-mouse Lumican (R&D Systems, Cat#: AF2745, 1:300)  Anti-mouse TRANCE/TNFSF11/RANKL (R&D Systems, Cat#: AF462, 1:300)  Anti-mouse INMT (Invitrogen, Cat#: PA-116585, 1:300)  Anti-Syrian hamster IgG AF594 (Jackson ImmunoResearch, Cat#: 307-585-003, 1:1000)  Anti-Syrian hamster IgG AF647 (Jackson ImmunoResearch, Cat#: 107-605-142, 1:1000)  Anti-rat IgG AF594 (Jackson Immunoresearch, Cat#: 712-585-153, 1:1000)  Anti-rabbit IgG AF488 (Jackson Immunoresearch, Cat#: 711-545-152, 1:1000)  Anti-rabbit IgG Cy3 (Jackson Immunoresearch, Cat#: 711-165-152, 1:1000)  Anti-rabbit IgG AF647 (Jackson Immunoresearch, Cat#: 711-605-152, 1:1000)  Anti-goat IgG AF594 (Jackson Immunoresearch, Cat#: 705-585-003, 1:1000)  Anti-goat IgG AF647 (Jackson Immunoresearch, Cat#: 705-607-003, 1:1000)  Streptavidin-AF647 (Jackson Immunoresearch, Cat#: 016-600-984, 1:1000)  Streptavidin-AF594 (Biolegend, Cat#: 405240, 1:1000)  4′,6-Diamidin-2-phenylindol (Invitrogen, Cat#: D1306, 1:1000) |
